# Portability of genomic predictions trained on sparse factorial designs across two maize silage breeding cycles

**DOI:** 10.1007/s00122-024-04566-4

**Published:** 2024-03-07

**Authors:** Alizarine Lorenzi, Cyril Bauland, Sophie Pin, Delphine Madur, Valérie Combes, Carine Palaffre, Colin Guillaume, Gaëtan Touzy, Tristan Mary-Huard, Alain Charcosset, Laurence Moreau

**Affiliations:** 1 Université Paris-Saclay, INRAE, CNRS, AgroParisTech, Génétique Quantitative et Evolution (GQE) - Le Moulon, 91190 Gif-Sur-Yvette, France; 2 UE 0394 SMH, INRAE, 2297 Route de l’INRA, 40390 Saint-Martin-de-Hinx, France; 3 Maïsadour Semences SA, 40001 Mont-de-Marsan Cedex, France; 4 RAGT2n, Genetics and Analytics Unit, 12510 Druelle, France; 5 Université Paris-Saclay, AgroParisTech, INRAE, UMR MIA Paris-Saclay, 91120 Palaiseau, France

## Abstract

**Key message:**

We validated the efficiency of genomic predictions calibrated on sparse factorial training sets to predict the next generation of hybrids and tested different strategies for updating predictions along generations.

**Abstract:**

Genomic selection offers new prospects for revisiting hybrid breeding schemes by replacing extensive phenotyping of individuals with genomic predictions. Finding the ideal design for training genomic prediction models is still an open question. Previous studies have shown promising predictive abilities using sparse factorial instead of tester-based training sets to predict single-cross hybrids from the same generation. This study aims to further investigate the use of factorials and their optimization to predict line general combining abilities (GCAs) and hybrid values across breeding cycles. It relies on two breeding cycles of a maize reciprocal genomic selection scheme involving multiparental connected reciprocal populations from flint and dent complementary heterotic groups selected for silage performances. Selection based on genomic predictions trained on a factorial design resulted in a significant genetic gain for dry matter yield in the new generation. Results confirmed the efficiency of sparse factorial training sets to predict candidate line GCAs and hybrid values across breeding cycles. Compared to a previous study based on the first generation, the advantage of factorial over tester training sets appeared lower across generations. Updating factorial training sets by adding single-cross hybrids between selected lines from the previous generation or a random subset of hybrids from the new generation both improved predictive abilities. The CDmean criterion helped determine the set of single-crosses to phenotype to update the training set efficiently. Our results validated the efficiency of sparse factorial designs for calibrating hybrid genomic prediction experimentally and showed the benefit of updating it along generations.

**Supplementary Information:**

The online version contains supplementary material available at 10.1007/s00122-024-04566-4.

## Introduction

Maize varieties are generally single-cross hybrids obtained by crossing two inbred lines that belong to complementary heterotic groups. The challenges for breeders are (i) selecting lines within each heterotic group that will be used as parents for the next generation and (ii) identifying the best single-cross hybrids among all possible ones in order to derive new varieties. The advent of Doubled-Haploid (DH) technology now enables the rapid production of numerous fully homozygous inbred lines each year. This large number of candidate lines produced each year in breeding programs makes generating and evaluating all potential single-cross hybrids practically undoable. To overcome this difficulty, conventional maize hybrid breeding schemes are typically divided into two stages. In the first stage (1), topcross hybrids are produced by crossing candidate lines from one heterotic group with a limited number of inbred lines from the complementary group, referred to as "testers". The performances of these topcross hybrids provide an estimation of the general combining abilities (GCA) of the candidate lines. In the second stage (2), the selected lines from stage 1 are crossed using a sparse factorial design to identify the best single-cross hybrid combinations. At this stage, the selection is performed on the GCA of the parental lines and the specific combining ability (SCA) of the pair of parental lines. Selecting lines based on a limited number of testers at stage 1 does not fully exploit the complementarity between the candidate lines from the two heterotic groups and can bias the line GCA estimation since the line GCA and its SCAs with the testers are confounded (Hallauer et al. 2010). Also, the two-stage process is time-consuming and requires extensive phenotyping (at least as many as the total number of candidate lines in both groups in stage 1).

Due to limited resources for phenotyping, predicting the performance of untested hybrids has been a critical objective in hybrid selection. Bernardo (1994) was the first to propose a marker-based model for plant hybrid performance prediction. He combined marker-based distances between parental lines of hybrids and the performances of a related set of single-crosses to predict GCAs and SCAs of non-phenotyped hybrids. This prediction model, which aims at predicting the value of unphenotyped individuals based on their marker-based relationship with a set of individuals both phenotyped and genotyped, is similar to the genomic best linear unbiased prediction (GBLUP) model (VanRaden 2008) that has been proposed more recently and is now widely used to perform genomic selection (GS) in plants and animal. Different other genomic prediction models have been proposed (see Meuwissen et al. 2001 seminal paper and Howard et al. 2022 for a review), all of them use molecular markers scored across the entire genome to predict the genetic values of genotyped individuals, referred to as the prediction set (PS), using individuals both phenotyped and genotyped, referred to as the training set (TRS). Since the pioneer work of Bernardo (1994), different prediction models adapted to hybrid value prediction have been proposed considering non-additive effects, either by modeling the GCA and SCA effects or the additive, dominance, and epistasis effects (Vitezica et al. 2013, 2017; Varona et al. 2018; González-Diéguez et al. 2021). Even if several experimental studies in maize have confirmed the efficiency of these GS models for predicting the value of single-cross hybrids (see review by Kadam and Lorenz (2018), and more recent papers such as Fristche-Neto et al. 2018; Wang et al. 2020; Kadam et al. 2021; Auinger et al. 2021; DoVale et al. 2022; Kamweru et al. 2023 and Heilmann et al. 2023),the relative interest of the different prediction models is still unclear. Besides the statistical model, various factors are known to affect genomic prediction accuracies, such as trait heritability, the number of markers, the size of the TRS, and the relationship between the TRS and the PS (see reviews: Kadam and Lorenz 2018; Isidro y Sánchez and Akdemir 2021; Merrick and Carter 2021; Kadam et al. 2021). In the context of hybrid prediction, in addition to these factors, the crossing design used to produce the TRS hybrids also affects prediction accuracy (Technow et al. 2014; Seye et al. 2020; Lorenzi et al. 2022; Melchinger et al. 2023; Melchinger and Frisch 2023).

In most studies, GS for hybrid value prediction has been considered in the second stage of the hybrid breeding scheme, i.e., by using as TRS hybrids between lines that have already undergone a selection based on their testcross values. To improve the efficiency of hybrid breeding schemes, Kadam et al. (2016) and Giraud (2016) proposed (1) to replace topcross evaluation in stage 1 with single-crosses issued from a sparse factorial design between unselected candidate lines from both groups and (2) to use GS to predict GCAs of all lines and SCA of all potential single-cross combinations. This makes it possible to perform selection in one stage instead of two. Both studies found good prediction accuracies for untested hybrids using factorial designs as TRS. The use of GS enables the consideration of very incomplete factorial designs composed of only one single-cross hybrid per line, which was not possible without marker information. At a fixed number of single-crosses, such factorial design makes it possible to evaluate twice as many candidate lines compared to a tester design composed of one tester per heterotic group, while allowing the estimation of each parental GCAs. Later, simulations and experimental studies have confirmed the potential of using sparse factorial instead of tester TRSs when predicting the same generation (Seye et al. 2020; Burdo et al. 2021; Lorenzi et al. 2022) and such schemes recently received a growing interest (Melchinger and Frisch 2023; Fritsche-Neto et al. 2023). For breeders, one important interest of GS is also to predict based on previous generations the hybrid values of a new generation of candidate lines prior to their phenotypic evaluation in order to guide seed production of only the most promising hybrids or to directly select the best lines as parents for the next generation without phenotyping them for their hybrid value (rapid cycling). Although a simulation work validated the advantage of factorial compared to tester TRSs to predict hybrid values across breeding cycles (Seye et al. 2020), further experimental validation is needed. From one cycle to the next, the average relatedness between the TRS and PS decreases and the joint effect of selection, drift, and recombination events change allele frequencies and the linkage disequilibrium between markers and QTLs, which decrease prediction accuracy if the TRS is not updated along cycles (Pszczola et al. 2012; Isidro y Sánchez and Akdemir 2021; Rio et al. 2022b). This raises questions about how to efficiently update the TRS to maximize prediction accuracy while minimizing phenotyping costs.

According to the literature, an ideal TRS should maximize the accuracy by maximizing the relationship between the TRS and PS (Zhong et al. 2009; Zhao et al. 2012; Technow et al. 2013) and minimizing the within TRS relationship to capture a large genetic variance (Pszczola et al. 2012; Isidro y Sánchez and Akdemir 2021). Different optimization criteria have been proposed to define the TRS (Rio et al. 2022b). Rincent et al. (2012) proposed optimizing the TRS by maximizing the mean of the coefficient of determination (CDmean) of contrasts between each unphenotyped PS individual and the target population mean. Numerous studies have shown that building the TRS using the CDmean significantly increases the accuracy of GS models relative to random sampling (Isidro y Sánchez and Akdemir 2021; Rio et al. 2022a, b; Fernández-González et al. 2023). In a breeding program, where genomic prediction is applied routinely, a large dataset from previous years of phenotyping is available for model training. One can wonder which phenotypic data from the previous generation(s) should be included in the TRS and which additional hybrids should be phenotyped to complete the existing TRS and achieve the highest prediction accuracy for the new generation with a given phenotyping effort. One option can be to add performances of new single-crosses between the parental lines selected to generate the new generation. This additional phenotyping can be done before it is possible to produce hybrids from the new generation and is in fact often done by breeders to identify potential new varieties. Another option is to wait until hybrids from the new generation can be produced and update the TRS with a subset of all potential hybrids. One idea could be to use the CDmean to optimize the choice of the individuals from the new generation to be phenotyped while considering the existing TRS comprising data from the previous generations. To our knowledge, this strategy has never been tested in this context.

The present study investigates the use and optimization of factorial TRS for genomic prediction of hybrid performance across breeding cycles. It relies on two breeding cycles of a reciprocal genomic selection scheme initiated from multiparental connected reciprocal populations generated in the flint and dent complementary heterotic groups. Data from the first cycle was already analyzed in previous studies (Giraud et al. 2017a, b; Seye et al. 2019) and have shown promising results in terms of genomic predictive abilities for replacing testcross evaluation by sparse factorial evaluations (Lorenzi et al. 2022). We present in this study results from a new breeding cycle that was produced and evaluated in a factorial design to: (i) estimate the genetic gain achieved after selection based on genomic predictions calibrated on a sparse factorial, (ii) assess the predictive ability in the new breeding cycle and compare different GS models, (iii) evaluate the efficiency of training GS models on a factorial design for predictions across breeding cycles and compare it to tester designs, (iv) investigate the benefit of different strategies to update the factorial TRS across cycles and optimize it to predict the new generation.

## Materials and methods

**Fig. 1:**
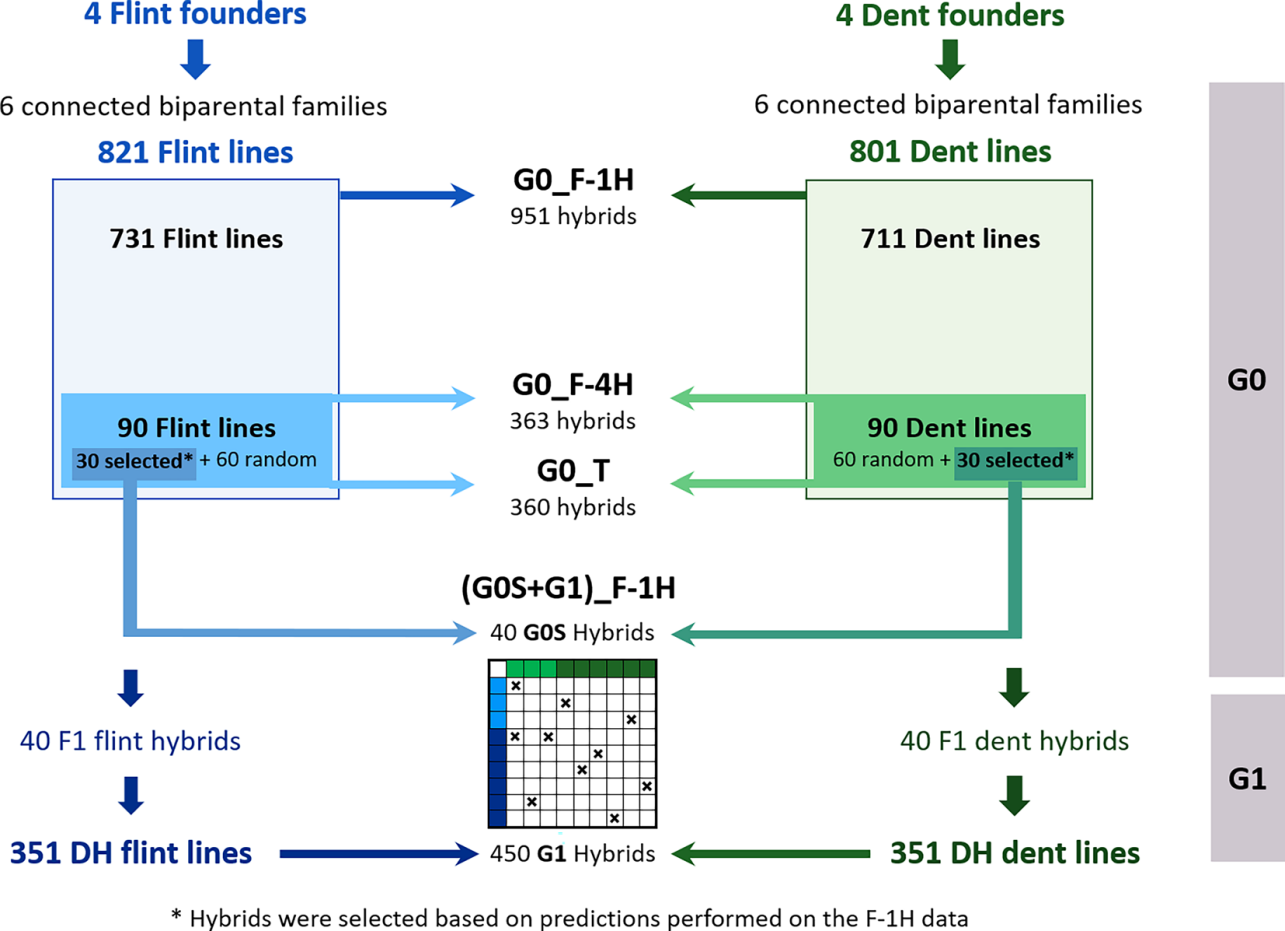
Hybrid experimental designs produced by crossing inbred lines from the initial generation (G0) and the inbred lines obtained after one cycle of selection (G1)

This study relies on data from a reciprocal breeding experiment aiming at improving the silage performance of maize single-cross hybrids produced between the dent and flint heterotic groups, the two main heterotic groups used for silage maize hybrids in Northern Europe. The experimental data comprises two breeding cycles, further called G0 and G1. Inbred lines from the G0 cycle were evaluated for hybrid performances in three experimental designs already analyzed in previous publications (Giraud et al. 2017a, b; Seye et al. 2019; Lorenzi et al. 2022). A summary of the G0 cycle production is provided below. The best G0 lines in each group were selected based on genomic predictions and intercrossed to produce the new breeding cycle (G1) we will focus on in this study. All experimental designs are described in Table 1 and Fig. 1.

**Table 1:** Description of all experimental designs used in this study

Years of phenotyping	Breeding cycle	Design	Name	Hybrids within the design^a^	References^c^
2013, 2014	G0	Factorial	G0_F-1H	G0R^a^	Giraud et al. 2017a, b; Seye et al. 2019; Lorenzi et al. 2022
2016, 2017	G0	Factorial	G0_F-4H	G0R + G0S^b^	Seye 2019; Lorenzi et al. 2022
		Tester	G0_T		
2019, 2020	G0 + G1	Factorial	(G0S + G1)_F-1H	G0S + G1	Current study

^a^G0R hybrids were produced by crossing two random lines from the G0 cycle

^b^G0S hybrids were produced by crossing two selected lines from the G0 cycle

^c^A reference was indicated for data that was already analyzed in previous studies

### Summary of the G0 plant material production and selection of the best candidate lines

Four founder lines were intercrossed in each group to derive six biparental families. In total, 822 flint lines and 802 dent lines were produced, further called G0 lines. The G0 lines were crossed to produce three experimental hybrid designs. The G0_F-1H was obtained by crossing the 822 flint lines to the 801 dent lines following a sparse factorial design, leading to 951 single-cross hybrids (on average, one line contributed to 1.2 hybrids). This experimental design was evaluated in France and Germany in 2013 and 2014 for silage performances (Giraud et al. 2017a, b; Seye et al. 2019). Then, 30 G0 lines were selected in each heterotic group based on genomic predictions of their GCAs trained on the G0_F-1H for an economic index combining silage yield, moisture content at harvest, and silage quality. The index used was: (DMY + 0.2 $\times$ (MS–33.5)) $\times$ MFU, where DMY, DMC and MFU stand for Dry Matter Yield, Dry Matter Content and Milk Fodder Unit, respectively (see below for a description of these traits). Additionally, 60 G0 lines (10 lines per family) were chosen randomly. These lines were used to create two other experimental designs. The G0_F-4H factorial design composed of 363 hybrids (on average, one line contributed to four hybrids) was produced by randomly crossing (i) the 30 G0 selected flint lines to the 30 G0 selected dent lines to produce 131 hybrids (further called “G0S hybrids”) and (ii) the 60 G0 random dent lines to the 60 G0 random flint lines leading to 232 hybrids (further called “G0R hybrids”). In parallel, the G0_T-F (and G0_T-D) tester design was produced by crossing the same 90 G0 flint (dent) lines from one group to two founder lines from the dent (flint) group used as testers. Together, the G0_T-F and G0_T-D tester designs were called G0 tester designs or G0_T. The G0_F-4H and the G0_T were evaluated jointly in eight trials in Northern France and Germany in 2016 and 1017 (Seye 2019; Lorenzi et al. 2022). In all trials, 18 hybrids were used as controls and evaluated twice: two commercial hybrids (LG30.275 and RONALDINIO) and 16 founder hybrids produced by crossing the four flint founder lines with the four dent founder lines.

### New breeding cycle (G1)

40 intragroup single-crosses were produced in each group by crossing the 30 selected G0 lines described above. 351 dent and 351 flint DH lines (G1) were derived from the 40 single-crosses in the dent and flint groups, respectively. The dent G1 lines were crossed with the flint G1 lines following a sparse factorial design to produce 442 G1 hybrids. Crosses were made at random with an average number of hybrids per line close to one. A new set of 47 G0S hybrids between the 30 G0 selected lines from each group was produced and evaluated jointly with the G1 hybrids, yielding a total of 489 hybrids further referred to as (G0S + G1)_F-1H. Hybrids were evaluated for two years in the North of France and Germany: three trials in 2019 and five in 2020. The same 18 control hybrids (two commercial and 16 founder hybrids) as in the G0 experiments were evaluated twice in each trial. 15% of the experimental hybrids were also replicated once at each location. The field experiments were laid out as augmented partially replicated designs (p-rep) (Williams et al. 2011). Each trial comprised 512 to 520 elementary plots distributed in 26 incomplete blocks of 20 plots. Each genotype was evaluated in 7 trials across 2019 and 2020 and was replicated in at least one trial. For each trial, repetitions were allocated to blocks to form an efficient incomplete block design using the DiGGer R package (Coombes 2009).

Hybrids were evaluated for 11 traits, four agronomical traits: silage yield (DMY in tons of dry matter per ha), dry matter content at harvest (DMC in % of fresh weight), female flowering date (DtSilk in days after January the first) and plant height (PH in cm) and seven silage traits for digestibility: milk fodder unit per kilogram of dry matter (MFU in MFU per kg) (Andrieu 1995) (computed using model 4.2), cell wall content of the harvested dry matter measured by the neutral detergent fiber content (NDF in % of dry matter), cell wall in vitro digestibility of the non-starch and non-soluble carbohydrates part of silage (DINAG in %) and cell wall in vitro digestibility of the non-starch, non-soluble carbohydrates and non-crude protein part of silage (DINAGZ in %), lignin, cellulose and hemicellulose contents in the cell wall NDF evaluated with the Goering and Soest (1970) method (LIGN, CELL, and HCELL in % of NDF). The DINAG and DINAGZ are two digestibility criteria first proposed by Argillier et al. (1995). The silage traits were predicted using Near Infrared Reflectance Spectrometry (NIRS) measured in the lab on silage powders or directly on fields during the harvest, depending on the trial.

Outlier observations were detected by examining raw data and considering field observations. They were treated as missing data. Subsequently, filters were applied to identify plots with standing counts below 80% of the median, and DMC below 25% or above 45%, which were also considered as missing data. Values of DINAG, DINAGZ, and MFU measured in two trials were inconsistent with those of other trials and were excluded from further analyses. Following quality control and filters, the percentage of missing data across all traits was 8%.

### Genotyping

The founder lines and the G0 parental lines were genotyped for 18,480 SNPs using a proprietary Affymetrix® array provided by Limagrain. The G1 parental lines were genotyped using a custom-made chip comprising a subset of 15,000 SNPs of the Illumina® MaizeSNP50 BeadChip (Ganal et al. 2011). Filters were applied for both G0 and G1 lines: markers with more than 20% of missing values within the dent and flint parental lines, markers with more than 5% of heterozygosity among the dent (flint) parental lines, and with Minor Allele Frequency (MAF) inferior to 5% were discarded. After quality control, only markers common to the two arrays were considered. 4,812 SNP polymorphic markers (in at least the flint or dent population) were retained for further analyses. Genotypes were encoded as 2 for the homozygotes with the reference allele which was the allele carried by the first individual of the data Table 1 for the heterozygotes, and 0 for the other homozygotes.

### Estimation of variance components and trait heritabilities

Variance components and trait heritabilities were estimated in the (G0S + G1)_F-1H design. Individual single-plot performances were corrected by the BLUPs of spatial effects predicted using the model defined in supplementary material File S1. Corrected data were then used to estimate variance components using the following model:

1 $${Y}_{hii^{\prime}jl}=\mu +{\lambda }_{l}+\left({\tau }_{h}+{\rho }_{lh}\right)\times {t}_{h}+\left[{\pi }_{j}+{H}_{h(i{i}^{^{\prime}})j}+H{\lambda }_{lh(i{i}^{^{\prime}})j}\right]\times \left(1-{t}_{h}\right)+{E}_{hi{i}^{^{\prime}}jl},$$

where ${Y}_{hii^{\prime}jl}$ is the phenotypic value corrected by spatial effects of hybrid $h$ of generation *j* produced by crossing the flint parental line $i$ and the dent parent line ${i}^{\prime}$ evaluated in trial $l$. $\mu$ is the intercept, ${\lambda }_{l}$ is the fixed effect of trial $l$, ${t}_{h}$ is an indicator function that distinguishes experimental hybrids (set to 0) from control hybrids (set to 1), ${\tau }_{h}$ is the fixed effect of control hybrids with 19 levels (2 for commercial hybrids + 16 for founder hybrids + one for non-control hybrids), ${\rho }_{lh}$ is the effect of the interaction between trial $l$ and control hybrid $h$, ${\pi }_{j}$ is the fixed effect of the generation with two levels (G0S or G1 hybrids). ${H}_{h({ii}^{\prime})j}$ is the random genetic effect of experimental hybrid $h$ of generation $j$, produced by crossing the flint line $i$ and the dent line $i^{\prime}$. ${H}_{h(i{i}^{\prime})j}$ is decomposed into its GCA and SCA components as follows:

$${H}_{h({ii}^{\prime})j}= {U}_{ij}+{U^{\prime}}_{i^{\prime}j}+{S}_{ii^{\prime}j}$$

where ${U}_{ij}$ (respectively ${U^{\prime}}_{i^{\prime}j}$) is the random GCA effect of the flint line $i$ (respectively dent line $i^{\prime}$) of generation $j$. We assume that ${U}_{ij}$ and ${U}_{i^{\prime}j}^{\prime}$ are independent and identically distributed (iid) within generation and follow a normal distribution: ${U}_{ij}\sim \mathcal{N}\left(0,{\sigma }_{{GCA}_{f}^{j}}^{2}\right)$ and ${U}_{i^{\prime}j}^{\prime}\sim \mathcal{N}\left(0,{\sigma }_{{GCA}_{d}^{j}}^{2}\right)$, respectively. ${\sigma }_{{GCA}_{f}^{j}}^{2}$ and ${\sigma }_{{GCA}_{d}^{j}}^{2}$ are the flint and dent GCA variances of generation $j$. ${S}_{kk^{\prime}}$ is the random SCA effect of the interaction between the parental lines $i$ and *i*′, with ${S}_{ii^{\prime}j}\sim \mathcal{N}\left(0,{\sigma }_{{SCA}^{j}}^{2}\right)$ ind with ${\sigma }_{{SCA}^{j}}^{2}$ being the SCA variance at generation $j$. $H{\lambda }_{lh({ii}^{\prime})j}$ is the genotype by trial interaction and is decomposed as follows:

$$H{\lambda }_{lh({ii}^{\prime})j}={\left(U\lambda \right)}_{ilj}+{\left({U}^{\prime}\lambda \right)}_{i^{\prime}lj}+{\left(S\lambda \right)}_{ii^{\prime}lj},$$

where ${(U\lambda )}_{ilj}$ and ${(U^{\prime}\lambda )}_{i^{\prime}lj}$ are the random effects of the flint GCA effect by trial interaction, respectively, dent GCA by trial interaction of generation $j$ and ${\left(S\lambda \right)}_{ii^{\prime}lj}$ is the random effect of the SCA by trial interaction of generation $j$. With ${(U\lambda )}_{ilj}\backsim \mathcal{N}\left(0,{\sigma }_{{GCA\times E}_{f}^{j}}^{2}\right)$, ${({U}^{\prime}\lambda )}_{i^{\prime}lj}\backsim \mathcal{N}\left(0,{\sigma }_{{GCA\times E}_{d}^{j}}^{2}\right)$ and ${\left(S\lambda \right)}_{ii^{\prime}lj}\backsim \mathcal{N}\left(0,{\sigma }_{{SCA\times E}^{j}}^{2}\right)$. ${\sigma }_{{GCA\times E}_{f}^{j}}^{2}$, ${\sigma }_{{GCA\times E}_{d}^{j}}^{2}$ and ${\sigma }_{{SCA\times E}^{j}}^{2}$ are the flint GCA by trial interaction variance, the dent GCA by trial variance and the SCA by trial interaction variance of generation $j$, respectively. ${E}_{hii^{\prime}jl}$ is the error term; we assume that the errors follow: ${E}_{hii^{\prime}jl}\sim \mathcal{N}\left(0,{\sigma }_{{E}_{l}}^{2}\right)$ and are iid within trial and independent between trials, ${\sigma }_{{E}_{l}}^{2}$ is the error variance of trial $l$. The different random effects of the model are assumed to be independent.

For each trait and each generation $j$ (G0S or G1), the percentage of genetic variance due to SCA was estimated (%), and broad-sense heritability was computed as follows:

$${H}_{j}^{2}=\frac{{\sigma }_{{H}_{j}}^{2}}{{\sigma }_{{H}_{j}}^{2}+\frac{{\sigma }_{H\times {E}_{j}}^{2}}{{n}_{{\text{site}}}}+\frac{{\sigma }_{{E}_{{\text{moy}}}}^{2}}{{n}_{{\text{rep}}}\times {n}_{{\text{site}}}}} ,$$

where ${\sigma }_{{H}_{j}}^{2}$ is the hybrid genetic variance of generation $j$ computed as ${\sigma }_{{H}_{j}}^{2}={\sigma }_{{{\text{GCA}}}_{f}^{j}}^{2}+{\sigma }_{{{\text{GCA}}}_{d}^{j}}^{2}+{\sigma }_{{{\text{SCA}}}^{j}}^{2}$, ${\sigma }_{H\times {E}_{j}}^{2}$ is the total genotype by trial variance of generation $j$ decomposed as: ${\sigma }_{H\times {E}_{j}}^{2}={\sigma }_{{{\text{GCA}}\times E}_{f}^{j}}^{2}+{\sigma }_{{{\text{GCA}}\times E}_{d}^{j}}^{2}+{\sigma }_{{{\text{SCA}}\times E}^{j}}^{2}$, and ${\sigma }_{{E}_{{\text{moy}}}}^{2}$ is the mean residual variance across all trials, ${n}_{{\text{site}}}$ is the average number of trials in which an hybrid has been evaluated and ${n}_{{\text{rep}}}$ is the average number of within trial replicates per hybrid across trials.

### Ls-means and genetic gain estimation

Least square-means (ls-means) of hybrids were computed over the eight trials. The model used was:

2 $${Y}_{{\text{hrl}}}^{*}=\mu +{\lambda }_{l}+{\gamma }_{h}+{E}_{{\text{hrl}}}$$

In this model, experimental hybrids and founder hybrids were considered jointly. ${Y}_{{\text{hrl}}}^{*}$ is the performance corrected by the spatial field effects of repetition $r$ of hybrid ℎ in trial $l$. $\mu$ is the intercept, ${\lambda }_{l}$ is the fixed effect of trial $l$, ${\gamma }_{h}$ is the fixed genetic effect of hybrid $h$. ${E}_{{\text{hrl}}}$ is the error term of environment $l$, with ${E}_{{\text{hrl}}} \sim \mathcal{N}\left(0,{\sigma }_{{E}_{l}}^{2}\right)$ iid within trial and independent between trials. All genomic predictions were performed on the ls-means thus obtained.

The founder hybrids were used as a reference for the initial unselected population. The observed genetic gain was computed as the difference between the performances of the founder hybrids and the experimental hybrids (either the G0S or the G1 hybrids). Then, we compared the observed to the predicted genetic gain estimated from the genomic predictions of hybrid values trained on the G0_F-1H.

### Pedigree-based best linear unbiased prediction (PBLUP) model

A prediction model based on the pedigree information (PBLUP) model was implemented and used as a benchmark compared to the GBLUP models. The model was:

3 $$y={1}_{n}.\mu +Zg+E,$$

where **y** is the vector of ls-means of the $n$ phenotyped hybrids, ${1}_{{\varvec{n}}}$ is a vector of $n$ ones and $\mu$ is the intercept.${\varvec{g}}$ is the vector of random hybrid effects, with ${\varvec{g}} \sim \mathcal{N}\left(0,{\varvec{K}}{\sigma }_{h}^{2}\right)$ where K is the pedigree kinship matrix computed on the hybrid population considering the founder lines as the base generation. The pedigree kinship matrix was computed with the recursive method presented in Mrode and Thompson (2005) using the AHGmatrix R-package (Amadeu et al. 2016). ${\sigma }_{h}^{2}$ is the hybrid variance. ${\varvec{Z}}$ is the corresponding incidence matrix. ${\varvec{E}}$ is the vector of error terms, with ${\varvec{E}}\sim \mathcal{N}(0,{\varvec{I}}{\sigma }_{E}^{2})$. The random effects are assumed to be independent.

### Genomic best linear unbiased prediction (GBLUP) models

Several GBLUP models were tested to evaluate the predictive ability within the G1 cycle. Two types of models can be distinguished: the GCA-models, which decompose the hybrid genetic effect into its parental GCAs and its SCA components and the G-models, which consider genetic effects defined based on the hybrid marker genotypes. GS models were fitted using the “MM4LMM” R-package (Laporte and Mary-Huard 2020; Laporte et al. 2022).

*GCA.1-model* Two GBLUP models were implemented for genomic predictions depending on the TRS design (factorial or tester). The model implemented on the factorial designs including SCA effects was:

4 $$y={1}_{n}.\mu +{Z}_{d} {g}_{{{\text{GCA}}}_{d}}+{Z}_{f}{g}_{{{\text{GCA}}}_{f}}+Z{g}_{{{\text{SCA}}}_{{\text{d}}f}}+E,$$

where ${\varvec{y}}$ is the vector of ls-means of the $n$ phenotyped hybrids, ${1}_{{\varvec{n}}}$ is a vector of $n$ ones and $\mu$ is the intercept. ${{\varvec{g}}}_{\mathbf{G}\mathbf{C}{\mathbf{A}}_{{\varvec{f}}}}$ (respectively ${{\varvec{g}}}_{\mathbf{G}\mathbf{C}{\mathbf{A}}_{{\varvec{d}}}}$) is the vector of random GCA effects of the ${n}_{f}$ flint parental lines (respectively ${n}_{d}$ dent lines), with ${{\varvec{g}}}_{\mathbf{G}\mathbf{C}{\mathbf{A}}_{{\varvec{f}}}} \sim \mathcal{N}\left(0,{{\varvec{K}}}_{{\mathbf{G}\mathbf{C}\mathbf{A}}_{{\varvec{f}}}}{\sigma }_{{{\text{GCA}}}_{f}}^{2}\right)$ (respectively ${{\varvec{g}}}_{\mathbf{G}\mathbf{C}{\mathbf{A}}_{{\varvec{d}}}} \sim \mathcal{N}\left(0,{{\varvec{K}}}_{{\mathbf{G}\mathbf{C}\mathbf{A}}_{{\varvec{d}}}}{\sigma }_{{GCA}_{d}}^{2}\right)$) where ${{\varvec{K}}}_{{\mathbf{G}\mathbf{C}\mathbf{A}}_{{\varvec{f}}}}$ (respectively ${{\varvec{K}}}_{{\mathbf{G}\mathbf{C}\mathbf{A}}_{{\varvec{d}}}}$) is the genomic relatedness matrix between the flint lines (respectively dent lines). The kinship matrix was computed for all the flint (dent) parental lines following method 1 from VanRaden (2008). ${\sigma }_{{{\text{GCA}}}_{f}}^{2}$ and ${\sigma }_{{{\text{GCA}}}_{d}}^{2}$ are the flint and dent GCA variances. ${{\varvec{g}}}_{\mathbf{S}\mathbf{C}{\mathbf{A}}_{\mathbf{d}{\varvec{f}}}}$ is the vector of SCA random effects of the $n$ hybrids, accounting for the interactions between the flint and dent parental lines, with ${{\varvec{g}}}_{\mathbf{S}\mathbf{C}{\mathbf{A}}_{\mathbf{d}{\varvec{f}}}} \sim \mathcal{N}\left(0,{{\varvec{K}}}_{{\mathbf{S}\mathbf{C}\mathbf{A}}_{\mathbf{d}{\varvec{f}}}}{\sigma }_{{{\text{SCA}}}_{{\text{d}}f}}^{2}\right)$ where ${{\varvec{K}}}_{{\mathbf{S}\mathbf{C}\mathbf{A}}_{\mathbf{d}{\varvec{f}}}}$ is the SCA kinship matrix of the hybrids (phenotyped or not) and ${\sigma }_{{SCA}_{df}}^{2}$ the SCA variance. The coefficient of the SCA kinship between two flint-dent hybrids produced from crossing parental lines $i$ to $j$ and parental lines *i*′ to *j*′ were computed as the product between the flint GCA kinship coefficient between lines $i$ and *i*′ and the dent GCA kinship coefficient between lines $j$ and *j*′ (Stuber and Cockerham 1966). ${{\varvec{Z}}}_{{\varvec{d}}}$, ${{\varvec{Z}}}_{{\varvec{f}}}$ and ${\varvec{Z}}$ are the corresponding incidence matrices. ${\varvec{E}}$ is the vector of error terms, with ${\varvec{E}}\sim \mathcal{N}\left(0,{\varvec{I}}{\sigma }_{E}^{2}\right)$. The different random effects are assumed to be independent.

The model implemented on the G0_T-F was:

5 $$y={1}_{n}.\mu +X\upsilon +{Z}_{f}{g}_{{{\text{GCA}}}_{f}}+Z{g}_{{\text{SCA}}t}+E,$$

where ${\varvec{y}}$ is the vector of ls-means of the $n$ phenotyped hybrids, ${1}_{{\varvec{n}}}$ is a vector of $n$ ones and $\mu$ is the intercept. ${\varvec{\upsilon}}$ is the vector of fixed effects of the two dent testers. ${{\varvec{g}}}_{\mathbf{G}\mathbf{C}{\mathbf{A}}_{{\varvec{f}}}}$ is the vector of random GCA effects of the ${n}_{f}$ flint parental lines, with ${{\varvec{g}}}_{\mathbf{G}\mathbf{C}{\mathbf{A}}_{{\varvec{f}}}} \sim \mathcal{N}\left(0,{{\varvec{K}}}_{{\mathbf{G}\mathbf{C}\mathbf{A}}_{{\varvec{f}}}}{\sigma }_{{{\text{GCA}}}_{f}}^{2}\right)$ where ${{\varvec{K}}}_{{\mathbf{G}\mathbf{C}\mathbf{A}}_{{\varvec{f}}}}$ is the genomic relatedness matrix between the flint lines and ${\sigma }_{{{\text{GCA}}}_{f}}^{2}$ is the flint GCA variance. ${{\varvec{g}}}_{\mathbf{S}\mathbf{C}\mathbf{A}{\varvec{t}}}$ is the vector of random effects of the interaction between the flint line and the dent testers, with ${{\varvec{g}}}_{\mathbf{S}\mathbf{C}\mathbf{A}{\varvec{t}}}\sim \mathcal{N}\left(0,{{\varvec{I}}}_{2}\otimes {{\varvec{K}}}_{\mathbf{G}\mathbf{C}\mathbf{A}\mathbf{f}\boldsymbol{ }}{\sigma }_{{\text{SCAt}}}^{2}\right)$ where ${\sigma }_{{\text{SCAt}}}^{2}$ is the SCA variance. The kinship matrix was computed for all the flint parental lines following method 1 from VanRaden (2008). ${\varvec{X}}$,${{\varvec{Z}}}_{{\varvec{f}}}$ and ${\varvec{Z}}$ are the corresponding incidence matrices. ${\varvec{E}}$ is the vector of error terms, with ${\varvec{E}} \sim \mathcal{N}\left(0,{\varvec{I}}{\sigma }_{E}^{2}\right)$. The different random effects are assumed to be independent. The same model was adapted and implemented on the G0_T-D.

*GCA.2-model* This GCA-model was defined following González-Diéguez et al. (2021), where the genetic effect is defined according to gamete origin. The fullest model for the factorial TRS was:

6 $$y={1}_{n}.\mu +{{Z}_{d}g}_{{A}_{d}}+{{Z}_{f}g}_{{A}_{f}}+{Zg}_{D}+{{Z}_{d}g}_{{{\text{AA}}}_{d}}+{{Z}_{f}g}_{{{\text{AA}}}_{f}}+{Zg}_{{{\text{AA}}}_{{\text{d}}f}}+E,$$

where ${\varvec{y}}$ is the vector of ls-means of the $n$ phenotyped hybrids, ${1}_{{\varvec{n}}}$ is a vector of $n$ ones and $\mu$ is the intercept. ${{\varvec{g}}}_{{{\varvec{A}}}_{{\varvec{f}}}}$ and ${{\varvec{g}}}_{{{\varvec{A}}}_{{\varvec{d}}}}$ are the vectors of the random additive effect from the flint and dent parental lines with ${{\varvec{g}}}_{{{\varvec{A}}}_{{\varvec{f}}}}\sim \mathcal{N}\left(0,{{\varvec{K}}}_{{{\varvec{A}}}_{{\varvec{f}}}}{\sigma }_{{A}_{f}}^{2}\right)$ and ${{\varvec{g}}}_{{{\varvec{A}}}_{{\varvec{d}}}}\sim \mathcal{N}\left(0,{{\varvec{K}}}_{{{\varvec{A}}}_{{\varvec{d}}}}{\sigma }_{{A}_{d}}^{2}\right)$, respectively. ${{\varvec{g}}}_{{\varvec{D}}}$ is the vector of random dominance effect with ${{\varvec{g}}}_{{\varvec{D}}}\sim \mathcal{N}\left(0,{{\varvec{K}}}_{{\varvec{D}}\boldsymbol{ }}{\sigma }_{D}^{2}\right)$, ${{\varvec{g}}}_{\mathbf{A}{\mathbf{A}}_{{\varvec{f}}}}$ is the vector of random additive-by-additive epistatic effect within the flint (resp. dent) population with  ${{\varvec{g}}}_{{\varvec{A}}{{\varvec{A}}}_{{\varvec{f}}}}\sim \mathcal{N}\left(0,{{\varvec{K}}}_{\mathbf{A}{\mathbf{A}}_{{\varvec{f}}}}{\sigma }_{{{\text{AA}}}_{f}}^{2}\right)$ (resp.${{\varvec{g}}}_{\mathbf{A}{\mathbf{A}}_{{\varvec{d}}}}\sim \mathcal{N}\left(0,{{\varvec{K}}}_{\mathbf{A}{\mathbf{A}}_{{\varvec{d}}}}{\sigma }_{{{\text{AA}}}_{d}}^{2}\right)$) and ${{\varvec{g}}}_{\mathbf{A}{\mathbf{A}}_{\mathbf{d}{\varvec{f}}}}$ is the vector of random additive-by-additive epistatic effect across the flint and dent populations ${{\varvec{g}}}_{{\varvec{A}}{{\varvec{A}}}_{{\varvec{d}}{\varvec{f}}}}\sim \mathcal{N}\left(0,{{\varvec{K}}}_{{\varvec{A}}{{\varvec{A}}}_{{\varvec{d}}{\varvec{f}}}}{\sigma }_{A{A}_{df}}^{2}\right)$. ${{\varvec{K}}}_{{{\varvec{A}}}_{{\varvec{f}}}}$, ${{\varvec{K}}}_{{{\varvec{A}}}_{{\varvec{d}}}}$, ${{\varvec{K}}}_{{\varvec{D}}\boldsymbol{ }},\boldsymbol{ }{{\varvec{K}}}_{\mathbf{A}{\mathbf{A}}_{{\varvec{f}}}\boldsymbol{ }},\boldsymbol{ }{{\varvec{K}}}_{\mathbf{A}{\mathbf{A}}_{{\varvec{d}}}}$ and ${{\varvec{K}}}_{\mathbf{A}{\mathbf{A}}_{\mathbf{d}{\varvec{f}}}}$ are, respectively, the flint additive, dent additive, dominance, additive-by-additive epistasis within the flint population, additive-by-additive epistasis within the dent population and the additive-by-additive epistasis across populations genomic relatedness matrices computed following González-Diéguez et al. (2021). ${\sigma }_{{A}_{f}}^{2}$,  ${\sigma }_{{A}_{d}}^{2}$, ${\sigma }_{D}^{2}$, ${\sigma }_{{{\text{AA}}}_{f}}^{2}$, ${\sigma }_{{{\text{AA}}}_{d}}^{2}$ and ${\sigma }_{{{\text{AA}}}_{{\text{d}}f}}^{2}$ are the corresponding variances. ${{\varvec{Z}}}_{{\varvec{f}}}$, ${{\varvec{Z}}}_{{\varvec{d}}}$ and ${\varvec{Z}}$ are the incidence matrices. ${\varvec{E}}$ is the vector of error terms, with ${\varvec{E}} \sim \mathcal{N}\left(0,{\varvec{I}}{\sigma }_{E}^{2}\right)$. The different random effects are assumed to be independent.

*G-model* This model was defined by Vitezica et al. (2017). It is based on the hybrid genotypes and does not account for the gamete origin (flint and dent parental origins). The fullest model considered for the factorial TRS was:

7 $$y={1}_{n}.\mu +{Zg}_{A}+{Zg}_{D}+{Zg}_{{\text{AA}}}+E,$$

where ${\varvec{y}}$ is the vector of ls-means of the $n$ phenotyped hybrids, ${1}_{{\varvec{n}}}$ is a vector of $n$ ones and $\mu$ is the intercept. ${{\varvec{g}}}_{{\varvec{A}}}$ is the vector of the random additive effect with ${{\varvec{g}}}_{{\varvec{A}}}\sim \mathcal{N}(0,{{\varvec{K}}}_{{\varvec{A}}\boldsymbol{ }}{\sigma }_{A}^{2})$, ${{\varvec{g}}}_{{\varvec{D}}}$ is the vector of random dominance effect with ${{\varvec{g}}}_{{\varvec{D}}}\sim \mathcal{N}(0,{{\varvec{K}}}_{{\varvec{D}}\boldsymbol{ }}{\sigma }_{D}^{2})$ and ${{\varvec{g}}}_{\mathbf{A}\mathbf{A}}$ is the vector of random additive-by-additive epistasis effect with ${{\varvec{g}}}_{{\varvec{A}}{\varvec{A}}}\sim \mathcal{N}(0,{{\varvec{K}}}_{\mathbf{A}\mathbf{A}\boldsymbol{ }}{\sigma }_{AA}^{2})$. ${{\varvec{K}}}_{{\varvec{A}}}$, ${{\varvec{K}}}_{{\varvec{D}}}$ and ${{\varvec{K}}}_{\mathbf{A}\mathbf{A}}$ are, respectively, the additive, dominance and additive-by-additive epistasis genomic relatedness matrices computed following Vitezica et al. (2017). ${\sigma }_{A}^{2}$, ${\sigma }_{D}^{2}$ and ${\sigma }_{{\text{AA}}}^{2}$ are the corresponding variances and **Z** is the incidence matrix. ${\varvec{E}}$ is the vector of error terms, with ${\varvec{E}} \sim \mathcal{N}(0,{\varvec{I}}{\sigma }_{E}^{2})$. The different random effects are assumed to be independent.

### Prediction scenarios

We defined three prediction scenarios to achieve three objectives: (i) assess the predictive ability of GS in the new generation by cross-validation and compare different GS models, (ii) evaluate the efficiency of a factorial design for predictions across breeding cycles and compare it to the tester designs, and (iii) investigate the benefit of different strategies to update the factorial TRS to predict the new generation, either by adding new phenotypic records for the parental lines of the new generation or by adding the new generation hybrids, and test the use of the CD mean criterion to optimize the TRS composition.

In Scenario 1, we evaluated the predictive ability within the new generation (G1 hybrids) and compared the efficiency of several GS models. This corresponds to a scenario when one wants to predict unobserved single-crosses hybrids using as TRS single-crosses derived from inbred lines of the same generation evaluated in the same environments. Such predictive ability will serve as a reference for the other scenarios. Cross-validations within the G1 hybrids were performed by training the GS model on 354 G1 hybrids (four-fifth) to predict the remaining 88 G1 hybrids (one-fifth). This process was repeated a hundred times. We compared three types of GBLUP models, namely the GCA.1-model, G-model, and GCA.2-model, to a benchmark PBLUP model. The GCA.1-model involved two nested models, with or without the SCA effect. For the GCA.2- and G-models, several nested models were tested by adding successively dominance and additive-by-additive genetic effects to additive effects. See Table 2 for the summary of all tested models.

**Table 2:** Definition of the genomic prediction models tested in Scenario 1

Models	Model code	Random genetic effects^a^	References
PBLUP		$g$	Henderson 1976
GCA.1	GCA	${g}_{{{\text{GCA}}}_{f}}+{g}_{{{\text{GCA}}}_{d}}$	VanRaden 2008
	GCA_SCA	${g}_{{{\text{GCA}}}_{f}}+{g}_{{{\text{GCA}}}_{d}}+{g}_{{\text{SCA}}}$	
GCA.2	GCA:A	${g}_{{A}_{f}}+{g}_{{A}_{d}}+r$	González-Diéguez et al. 2021
	GCA:AD	${g}_{{A}_{f}}+{g}_{{A}_{d}}+{g}_{D}$	
	GCA:A(AAdf)	${g}_{{A}_{f}}+{g}_{{A}_{d}}+{g}_{{{\text{AA}}}_{{\text{d}}f}}$	
	GCA:AD(AAdf)	${g}_{{A}_{f}}+{g}_{{A}_{d}}+{g}_{D}+{g}_{{{\text{AA}}}_{{\text{d}}f}}$	
	GCA:AD(AAf)(AAd)(AAdf)	${g}_{{A}_{f}}+{g}_{{A}_{d}}+{g}_{D}+{g}_{{{\text{AA}}}_{f}}+{g}_{A{A}_{d}}+{g}_{A{A}_{df}}$	
G	G:A	${g}_{A}$	Vitezica et al. 2017
	G:AD	${g}_{A}+{g}_{D}$	
	G:A(AA)	${g}_{A}+{g}_{{\text{AA}}}$	
	G:AD(AA)	${g}_{A}+{g}_{D}+{g}_{{\text{AA}}}$	

^a^The list of the random genetic effects considered in the GCA models correspond to: dent GCA ${(g}_{{{\text{GCA}}}_{d}}$), flint GCA ${(g}_{{{\text{GCA}}}_{f}}$), SCA ${(g}_{{\text{SCA}}}$), intragroup additive-by-additive epistasis for the dent ${(g}_{{{\text{AA}}}_{d}}$) and flint group ${(g}_{A{A}_{f}}$), and intergroup additive-by-additive epistasis ${(g}_{{{\text{AA}}}_{{\text{d}}f}}$) effects. In the G models, random genetic effects correspond to: additive ${(g}_{A}$), dominance ${(g}_{D}$) and additive-by-additive epistasis ${(g}_{{\text{AA}}}$) effects

Scenario 2 evaluated the efficiency of training a prediction model on the previous generation to predict the next generation hybrids. To this end a GBLUP model (GCA.1) was trained on the G0 generation to predict the next one (G1). In Scenario 2a, we evaluated the efficiency of the incomplete factorial TRS (G0_F-1H) to predict G1 hybrids. We assessed the prediction stability across breeding cycles by comparing the predictive abilities obtained for the G1 hybrids to the one obtained for the G0S hybrids evaluated in the same experiments. The GCA.1 model was used to perform predictions. In Scenario 2b, we compared the efficiency of factorial and tester TRSs from the G0 cycle to predict the G1 cycle. The GCA.1 models (4.1) or (4.2) were trained on the G0_F-4H (363 hybrids) or the tester designs (360 hybrids) to predict G1 hybrids. We investigated the impact of the TRS on hybrid selection through the correlation between the GCA BLUPs predicted using the factorial and the ones obtained using the tester designs. In addition, to compare the similarity of selection between the different approaches (based on phenotypic evaluations (ls-means) or genomic predictions (BLUPs) trained on the factorial or the tester designs), the coincidence of selection was computed for each trait. For each pair of approaches, it corresponds to the percentage of common hybrids that would be selected by the two approaches at a given selection rate (%). This coincidence of selection was computed for different selection rates. As in Lorenzi et al. (2022), we sampled hybrids in the tester designs to evaluate the impact of the number of testers used in the TRS. In this Scenario 2b’, each tester TRS was composed of 180 hybrids produced by crossing in each group: (i) 90 lines to one tester (180 lines in total): since there were two testers in each group, there were four possible tester combinations, referred to as 1T-180H-180L- followed by the names of the testers, (ii) 45 lines to one tester and the 45 other lines to the other tester, referred to as 2T-180H-180L, (iii) the same 45 lines to two testers referred to as 2T-180H-90L. We compared these tester TRSs to a factorial TRS by sampling 180 hybrids from the G0_F-4H in a random and balanced manner between families to maximize the number of lines. This factorial TRS comprised 180 hybrids representing 170 lines (one line contributed to 2.1 hybrids on average) and was called F-180H-170L. In Scenario 2b’, all the TRSs were sampled ten times except for the one-tester designs that were sampled only once.

Scenario 3 investigated TRS optimization across breeding cycles. One key question is to evaluate the benefit of updating a TRS based on a sparse factorial between unselected lines from the previous generation by either (i) further evaluating hybrids between parents of the new generation (G0S) or (ii) adding hybrids from the new generation (G1) or (iii) combining the two types of hybrids (G0S + G1). In Scenario 3a, we evaluated the benefit of updating the TRS across cycles by adding either G0S or/and G1 hybrids to the initial G0R TRS. Several TRSs were sampled and compared to cross-validations within the G1 hybrids (Scenario 1). To assess the benefit of adding G0S hybrids to the initial G0R TRS, we compared TRSs only composed of G0R hybrids with the same TRSs to which 132 G0S hybrids from the G0_F-4H design were added. To evaluate the benefit of updating G0 TRSs with G1 hybrids, we added from 0 to 354 randomly sampled G1 hybrids to G0 TRSs. One-fifth of the G1 hybrids (88 hybrids) were predicted using the GCA.1 model. The mean predictive ability over 100 replicates was computed for each TRS. In Scenario 3b, our objective was to maximize the predictive ability of the G1 hybrids by optimizing a priori the G1 hybrid subset used to update the initial G0 TRS using only G1 line genotypes. We considered the CDmean proposed by Rincent et al. (2012). We used a heritability of 0.7, corresponding to the average heritability of our traits, to compute the value for the shrinkage parameter $\lambda$ and the additive covariance kinship between hybrids defined by Vitezica et al. (2017). Two optimization strategies were considered and compared to random sampling. For both strategies, we optimized the mean of the CD of contrasts between each non-phenotyped G1 hybrid (PS) and the mean of the G1 hybrids. In the first strategy (CDmean1), the G1 hybrid set was optimized without considering the marker information on the G0 hybrids. The additive kinship considered to compute expected CDmeans only included the 442 G1 hybrids. In the second strategy (CDmean2), the optimization of the G1 was performed by also considering information on the G0 hybrids: the additive kinship was computed for all 1802 hybrids from both generations (1360 G0 + 442 G1). The procedure was performed in both scenarios with four sampling sizes for the G1 hybrids (50,100, 200, and 300) and replicated a hundred times each. For each optimized set, all G0 hybrids plus the chosen G1 hybrids were used as TRS to predict the remaining G1 hybrids, used as VS. Predictions were performed using the GCA.1 model.

### Predictive ability and statistical tests

In all scenarios, the predictive ability was computed as Pearson’s correlation between predicted hybrid values and hybrid ls-means. Different statistical tests were performed depending on the scenario to test the significance of differences between predictive abilities. In Scenario 1, paired t-tests were performed with a risk level α = 0.05, and a Bonferroni correction (multiple comparison correction) was applied per trait. In Scenario 2b, Williams tests (Williams 1959) were performed with a risk level *α* = 0.05 using the “r.test” function of the psych R-package (Revelle 2021). In Scenario 2b’, t-tests with a risk level *α* = 0.05 were performed, and a Bonferroni correction was applied per trait. For all scenarios, computations were performed in the R statistical environment (R Core Team 2020), and variance decomposition and GS models were fitted using the “MM4LMM” R-package (Laporte and Mary-Huard 2020; Laporte et al. 2022).

## Results

For clarity purposes, results on the four main traits of interest (DMY, DMC, DtSilk, and MFU) are presented in the following. The results on the 11 studied traits are shown in supplementary materials.

### Variance components and broad-sense heritability (H2) at the phenotypic level without marker information

Broad-sense heritabilities (H2) were medium to high (Table 3). They ranged from 0.56 (MFU) to 0.93 (DtSilk) for G0S hybrids and from 0.62 (MFU) to 0.94 (DtSilk) for G1 hybrids. Large and significant genetic variances were observed for all traits (Tables 3, S1) with no clear differences between G0S and G1 hybrids. The main part of the genetic variance was due to GCA. The proportion of genetic variance due to SCA ranged from 0% (DMC) to 30% (MFU) for the G0S and from 0% (DMY) to 10% (DMC) for the G1. ${\sigma }_{{{\text{GCA}}}_{f}}^{2}$ was always larger than ${\sigma }_{{{\text{GCA}}}_{d}}^{2}$ except for G0S hybrids for DMC. Non-null GCA by trial variances were observed for G0S and G1 hybrids, but were lower than the GCA variances. For the G0S, ${\sigma }_{{\text{SCA}}}^{2}$ was larger than ${\sigma }_{{\text{SCA}}xE}^{2}$ for all traits. For G1 hybrids, ${\sigma }_{{\text{SCA}}xE}^{2}$ was larger than ${\sigma }_{{\text{SCA}}}^{2}$, except for DtSilk.

**Table 3:** Broad-sense heritability (H2), percentage of genetic variance assigned to SCA variance (%SCA) and variance components estimated on phenotypic data corrected for spatial effects for the (G0S + G1)_F-1H without marker information

	DMY (t/ha)		DMC (%)		DtSilk (days)		MFU (MFUx10^2^/kg)	
	G0S	G1	G0S	G1	G0S	G1	G0S	G1
${\sigma }_{{{\text{GCA}}}_{f}}^{2}$	0.50(0.24)^d^	0.31(0.05)	0.46(0.28)	1.47(0.21)	1.63(0.71)	2.06(0.29)	0.44(0.24)	0.49(0.09)
${\sigma }_{{{\text{GCA}}}_{d}}^{2}$	0.13(0.18)	0.25(0.05)	1.42(0.51)	0.73(0.21)	1.57(0.65)	1.41(0.27)	0.00	0.36(0.09)
${\sigma }_{{\text{SCA}}}^{2}$	0.14(0.18)	0.00	0.00	0.25(0.20)	0.03(0.27)	0.05(0.22)	0.19(0.13)	0.00(0.09)
${\sigma }_{{{\text{GCA}}}_{f}\times E}^{2}$	0.07(0.08)	0.05(0.05)	0.00	0.11(0.05)	0.41(0.16)	0.17(0.07)	0.27(0.17)	0.20(0.07)
${\sigma }_{{{\text{GCA}}}_{d}\times E}^{2}$	0.12(0.09)	0.07(0.04)	0.39(0.13)	0.31(0.05)	0.05(0.12)	0.09(0.06)	0.06(0.12)	0.13(0.07)
${\sigma }_{{\text{SCA}}\times E}^{2}$	0.00	0.23(0.07)	0.00	0.00	0.00	0.24(0.11)	0.00	0.11(0.09)
${\sigma }_{E}^{2}$^a^	0.31(0.05)−1.40(0.12)		0.57(0.07)−3.62(0.27)		0.64(0.09)−2.33(0.20)		0.12(0.02)−7.97(0.55)	
%SCA^b^	19	0	0	10	1	1	30	0
H^2c^	0.87	0.80	0.90	0.92	0.93	0.94	0.56	0.62

^a^Minimum and maximum residual variance across all environments

^b^Percentage of SCA variance computed as $\frac{{{\varvec{\sigma}}}_{\mathbf{S}\mathbf{C}\mathbf{A}}^{2}}{{{\varvec{\sigma}}}_{\mathbf{G}\mathbf{C}{\mathbf{A}}_{{\varvec{d}}}}^{2}+{{\varvec{\sigma}}}_{\mathbf{G}\mathbf{C}{\mathbf{A}}_{{\varvec{f}}}}^{2}+{{\varvec{\sigma}}}_{\mathbf{S}\mathbf{C}\mathbf{A}}^{2}}\times 100$

^c^Broad-sense heritability

^d^Standard error in brackets

### Ls-means and genetic gain

On average, G0S and G1 hybrids performed similarly (Table 4). Compared to the 16 founder hybrids, which are representative of the performance of the unselected G0 hybrids (G0R), G0S and G1 hybrids showed a gain in performance for DMY (+ 1.55 t/ha for G0S and + 1.52 t/ha for G1). This gain was associated with a later DtSilk (+ 1.83 days for G0S and + 1.90 days for G1), a lower DMC (− 0.77% for G0S and − 0.67% for G1), and a lower MFU (− 2.11 MFUx10^2^/kg for G0S and − 2.15 MFUx10^2^/kg for G1). The observed genetic gain for DMY was similar to the predicted one based on the genomic predictions trained on the G0_F-1H design. However, for DMC, DtSilk, and MFU, the observed response to selection was higher in absolute value than the predicted one.

**Table 4:** Performances (ls-means) of commercial, founder and experimental hybrids (G0S and G1 hybrids) and genetic gain of the experimental hybrids compared to the founder hybrids corresponding to the (G0S + G1)_F-1H design

	Hybrid type	Component	DMY (t/ha)	DMC (%)	DtSilk (days)	MFU (MFUx10^2^/kg)
Ls-means	Commercial	Mean	**17.96** (17.18–18.74)^b^	**34.69** (34.37–35.00)	**201.89** (200.27–203.50)	**95.54** (95.34–95.74)
		Sd^c^	1.10	0.45	2.28	0.28
	Founder	Mean	**15.80** (14.27–17.47)	**34.10** (30.53–37.48)	**203.14** (199.80–206.16)	**95.28** (91.13–98.14)
		Sd	0.84	1.94	1.50	2.05
	G0S	Mean	**17.35** (14.82–18.89)	**33.33** (30.54–37.13)	**204.97** (201.80–208.79)	**93.17** (89.04–97.06)
		Sd	0.92	1.40	1.82	1.56
	G1	Mean	**17.33** (14.33–19.72)	**33.43** (29.39–39.18)	**205.04** (197.98–211.40)	**93.13** (87.59–101.36)
		Sd	0.85	1.63	1.97	1.71
Genetic gain^a^	G0S		1.55	−0.77	1.83	− 2.11
	G1		1.52	−0.67	1.90	− 2.15
Predicted genetic gain^d^	G0S		1.45	−0.33	1.18	− 1.35
	G1		1.41	−0.28	0.91	− 1.14

^a^Genetic gain computed as the difference between the mean performance of the experimental hybrids and the founder hybrids

^b^Mean performance in bold, and minimum and maximum mean performance in brackets

^c^Standard deviation of the ls-means of the experimental hybrid performances

^d^Predicted genetic gain based on genomic predictions trained on the G0_F-1H

### Scenario 1-predictive ability within the G1 cycle and GS model comparison

**Fig. 2:**
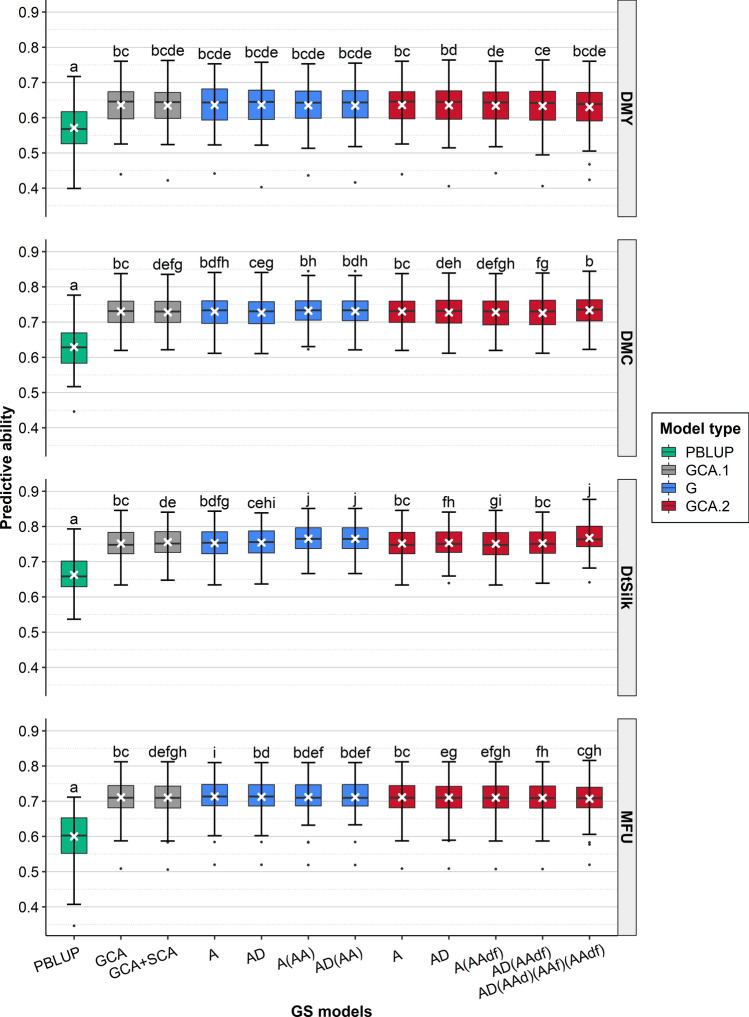
Predictive abilities obtained by cross-validations within the 442 G1 hybrids using different prediction models (PBLUP, GCA.1, GCA.2 or G models) in Scenario 1. The mean predictive ability over the 100 replicates is represented by a white cross. Significant differences (as obtained by paired t-tests at a level risk *α* = 0.05) are indicated with letters: two different letters indicate a significant difference and at least one common letters indicate no significant difference

We assessed the predictive ability in the new breeding cycle using cross-validations among G1 hybrids (Fig. 2). GBLUP predictive abilities of the new generation were high for all traits, ranging from 0.63 (DMY) to 0.76 (DtSilk) when considering the best GBLUP model. All GBLUP models significantly outperformed the PBLUP model (differences between the worst GBLUP model and the PBLUP ranged from 0.07 (DMY) to 0.11 (MFU)). Differences among GBLUP models were sometimes significant but minor (< 0.01), showing that models were equivalent and that adding non-additive effects had little effect.

### Scenario 2-efficiency of a factorial TRS for predictions across breeding cycles and comparison with tester TRSs

**Fig. 3:**
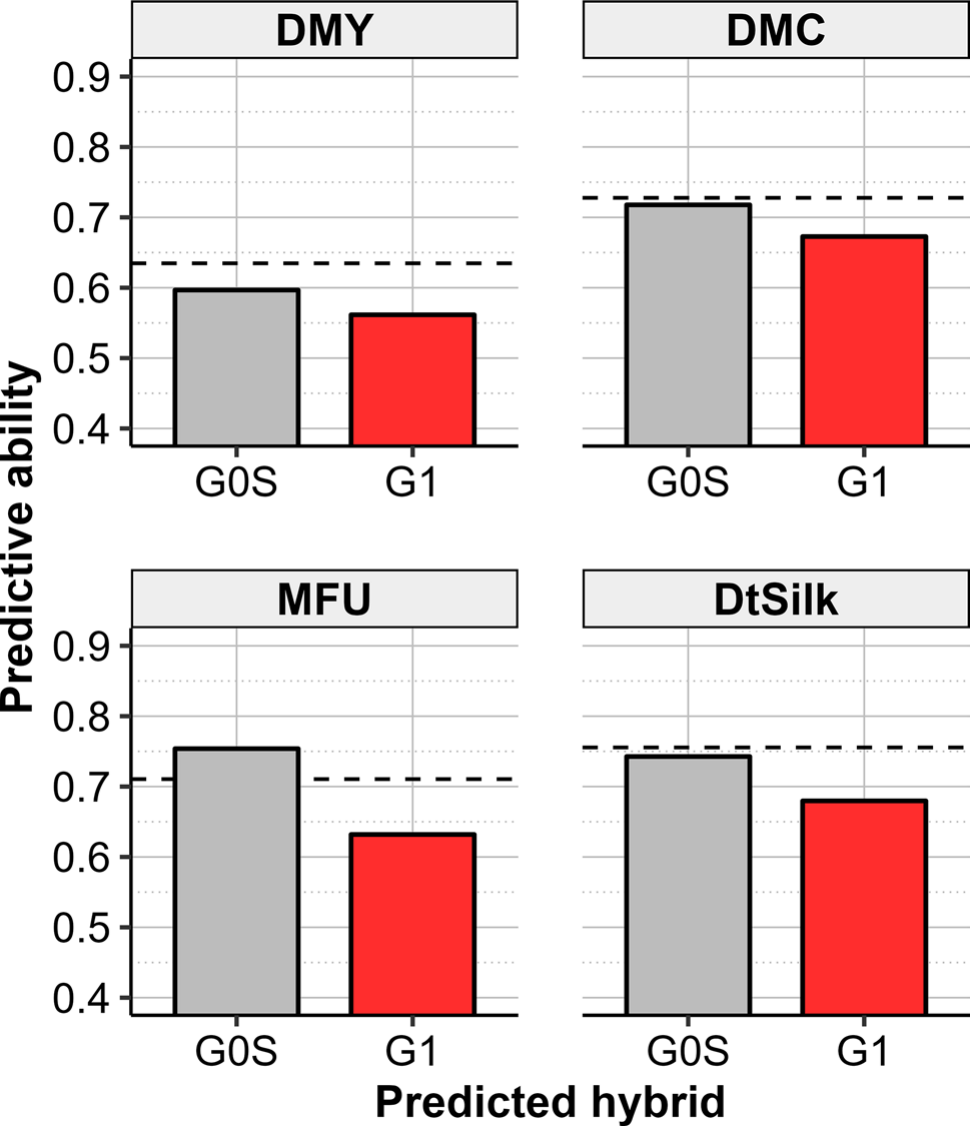
Predictive abilities obtained in Scenario 2a when training the GS model on the G0_F-1H design (951 hybrids) to predict the 47 G0S hybrids or the 442 G1 hybrids. The dotted line corresponds to the mean predictive ability over 100 replicates of cross-validations within the 442 G1 hybrids

In Scenario 2a, we compared the ability of the G0_F-1H TRS to predict the same generation (G0S hybrids) or the new generation (G1 hybrids). Predictive abilities were high for all traits (ranging from 0.56 for DMY to 0.67 for DtSilk for G1 hybrids and from 0.60 for DMY to 0.75 for MFU for G0S hybrids) (Fig. 3). As expected, predictive abilities were higher for G0S hybrids (hybrids from the same generation as the TRS hybrids) than for G1 hybrids for all traits. Lower predictive abilities were obtained when training on the G0_F-1H compared to those obtained by cross-validations within G1 hybrids (Fig. 3).

**Fig. 4:**
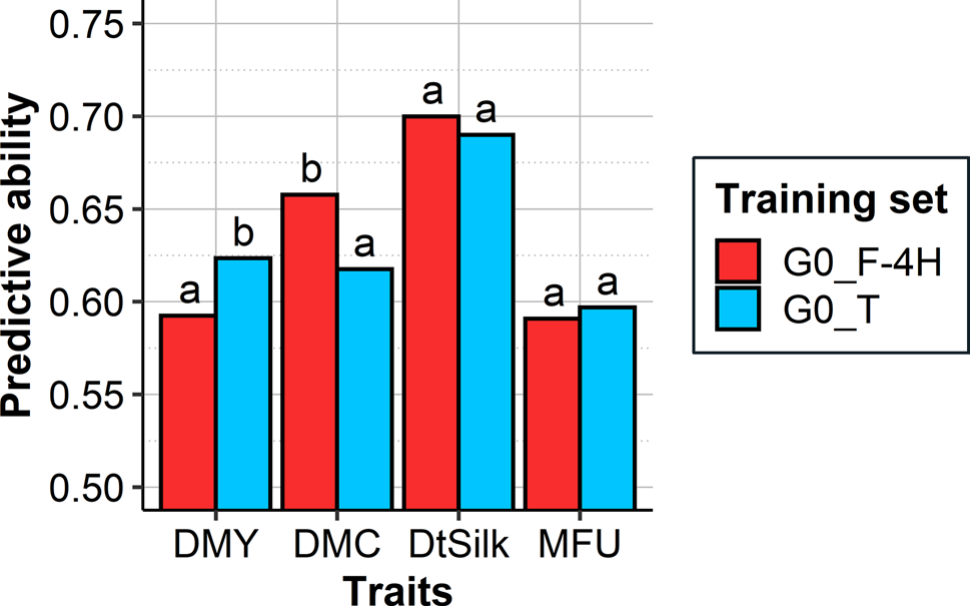
Predictive abilities obtained for the G1 hybrids (442) by training the GS model on the G0_F-4H (363) or the G0_T (360) TRSs in Scenario 2b. Williams tests were performed (*α* = 0.05) and significant differences were indicated with letters: two different letters indicate a significant difference and at least one common letters indicate no significant difference

In Scenario 2b, we compared predictive abilities obtained using either the G0_F-4H (363 hybrids) or the G0 tester designs (360 hybrids) as TRS to predict all G1 hybrids (442 hybrids) (Fig. 4). They ranged from 0.59 (DMY and MFU) to 0.70 (DtSilk) when training on the G0_F-4H and from 0.60 (MFU) to 0.69 (DtSilk) when training on the G0 tester designs. Across the 11 traits, training on the G0_F-4H or the G0 tester designs gave equivalent predictive abilities except for four traits: the G0_F-4H design significantly outperformed the G0 tester designs for DMC and PH, and the G0 tester designs significantly outperformed the G0_F-4H design for DMY and CELL (Fig. S1). The GCA BLUPs of the G1 lines predicted using the G0_F-4H or the G0 tester designs as TRS were highly correlated. They ranged from 0.85 (DMC) to 0.94 (MFU) for the dent G1 lines and from 0.84 (DMY) to 0.94 (DtSilk) for the flint G1 lines, and from 0.87 (DMY, DMC) to 0.91 (DtSilk) for G1 hybrids (Table S4). The coincidence of selection for genomic predictions between the factorial and the tester TRS of the top 5% of hybrids was 52% for DMY, 61% for DMC, 65% for DtSilk, and 39% for MFU (Fig. S2), which indicates that the single-cross hybrid sets selected by the two approaches are not identical. To assess if one of the two approaches identified a higher proportion of the best-phenotyped hybrids, we compared the proportion of the top 5% hybrids identified based on the factorial or tester TRS to the top 5% phenotyped hybrids. For DMY, the major trait of interest in our study, the factorial design identified a higher proportion of the best-phenotyped hybrids compared to the tester designs.

**Fig. 5:**
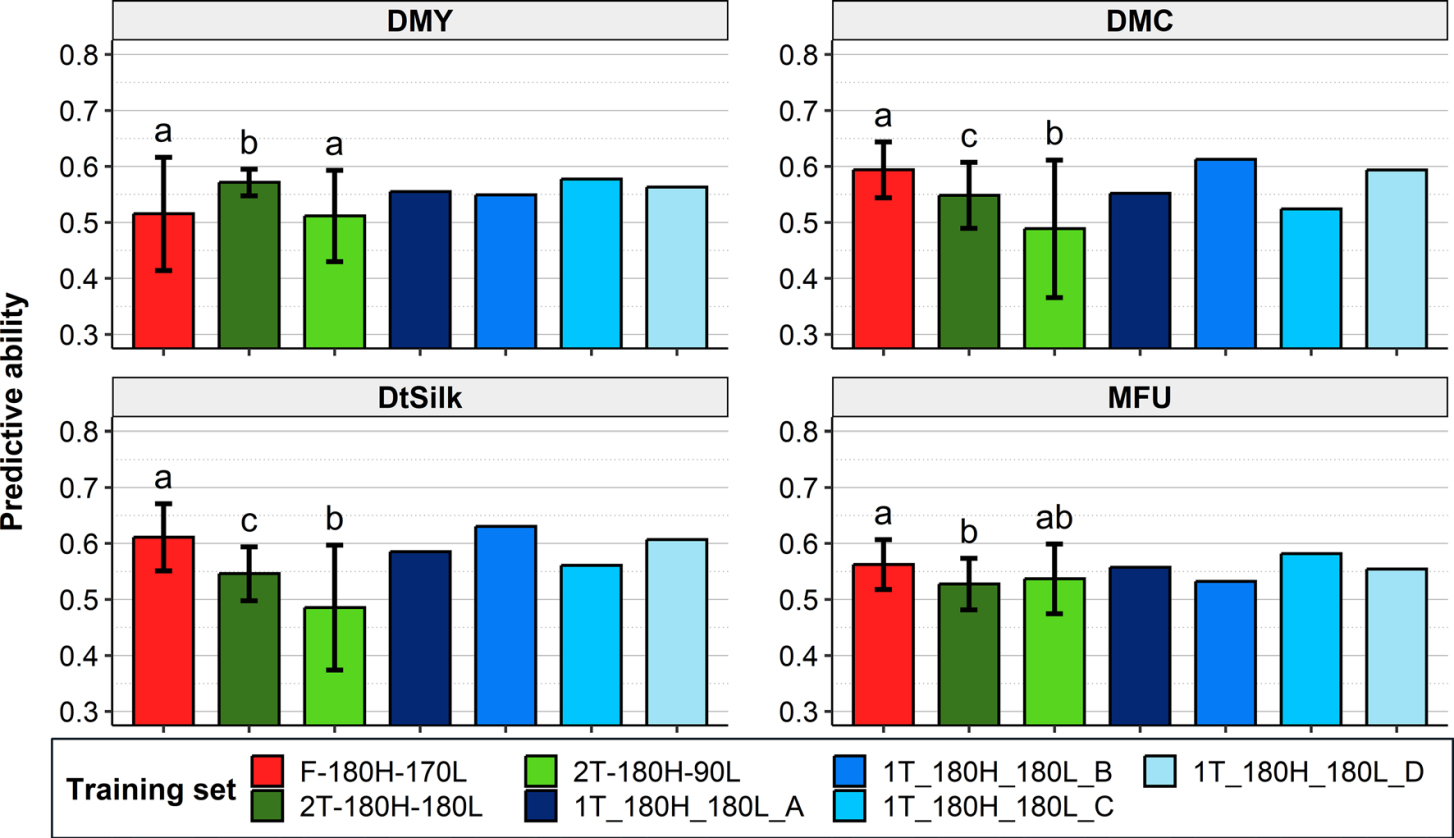
Predictive abilities obtained in Scenario 2b’ by training the GS model on 180 hybrids issued from tester-based or factorial TRSs to predict the G1 hybrids (442). The different tester-based TRSs correspond to: 90 lines crossed to one tester (1T-180H-180L-A, 1T-180H-180L-B, 1T-180H-180L-C, 1T-180H-180L-D), 90 lines crossed to two testers (2T-180H-180L), 45 lines crossed to two testers (2T-180H-90L). The factorial design (F-180H-152L) corresponds to the crosses of 76 flint lines with 76 dent lines. The sampling was repeated 10 times and t-tests (*α* = 0.05) were performed for the F-180H-170L, 2T-180H-180L and 2T-180H-90L. Significant differences were indicated with letters: two different letters indicate a significant difference and at least one common letters indicate no significant difference

In Scenario 2b’, we investigated the efficiency of different G0 tester design compositions to predict G1 hybrids (442 hybrids) at the same number of hybrids (180) and compared them with a factorial design of same size (Fig. 5). Predictive abilities varied between the four one-tester TRS ranging from 0.005 (DMC) to 0.048 (DMY), and the best one-tester TRS depended on the trait. The best two-tester TRS maximized the number of evaluated candidate lines by crossing more lines each to a different tester (2T-180H-180L) and usually outperformed the worst one-tester TRS. The F-180H-170L factorial TRS was equivalent to or outperformed the tester TRS except for DMY. On average, over the 11 traits, the F-180H-170L TRS gave the highest predictive abilities (Figs. 5, S3).

### Scenario 3a-benefit of updating the factorial TRS across breeding cycles

**Fig. 6:**
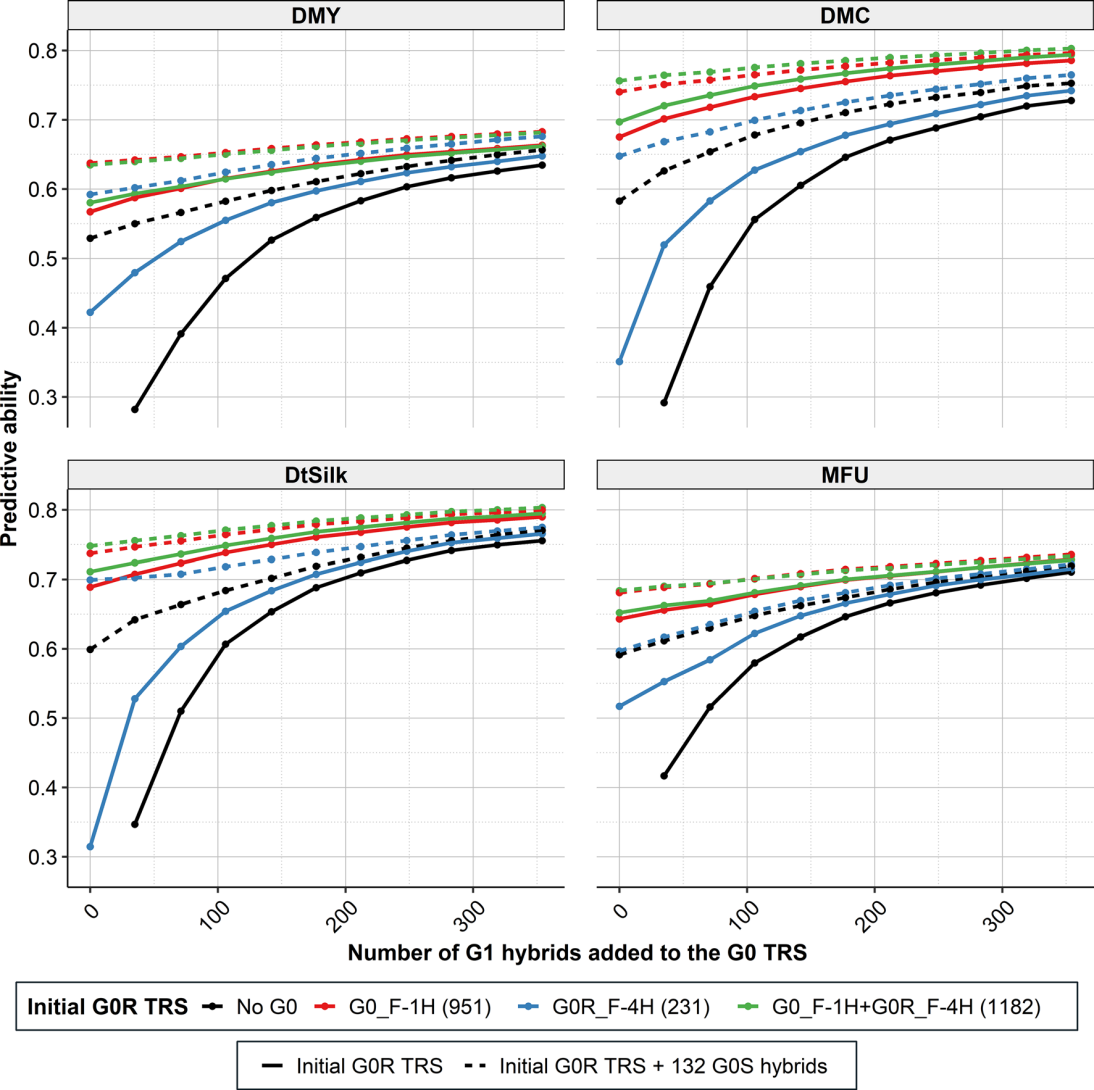
Predictive abilities obtained in Scenario 3a when predicting one-fifth of the G1 hybrids (88) using different TRSs: G0R hybrids (in solid colored lines) completed by 132 G0S hybrids (in doted colored lines) and *m* G1 hybrids (with *m* ranging from 0 to 354 from the left to the right of each graph). The mean predictive ability over 100 replicates is represented by a dot for each TRS. The number of hybrids in the initial G0R TRSs is indicated between brackets in the figure legend

To evaluate the benefit of updating the TRS across breeding cycles, four TRS strategies were evaluated based on their ability to predict G1 hybrids: (i) training on G0R only (G0_F-1H, G0R_F-4H or G0_F-1H + G0R_F-4H), (ii) training on G0R plus 132 hybrids between G0 selected lines (G0S), (iii) training on G0R plus a subset *m* of hybrids from the new generation (G1 hybrids), and (iv) training on G0R plus 132 G0S hybrids and *m* G1 hybrids, with *m* ranging from 1 to 354 (Fig. 6). The four TRS strategies were also compared to cross-validations within the G1 hybrids. The best G0R TRS (G0_F-1H + G0R_F-4H) was also the largest one (1183 hybrids) with predictive abilities ranging from 0.69 (MFU) to 0.76 (DMC and DtSilk), which were equivalent or higher than the ones obtained with a TRS composed of 354 G1 hybrids.

Adding 132 G0S hybrids to the initial G0R TRSs (G0_F-1H, G0R_F-4H, or G0_F-1H + G0R_F-4H) increased predictive abilities (with a gain on average of 0.10 for DMY, 0.14 for DMC, 0.16 for DtSilk and 0.05 for MFU). The largest gain in predictive ability was observed for the G0R_F-4H TRS, which was also the smallest G0R TRS (232 hybrids), with gains ranging from 0.08 (MFU) to 0.39 (DtSilk). There was always a gain in predictive ability when adding G1 hybrids to the TRS, whether composed of G0R or of G0R and G0S hybrids. As expected, the gain increased with the number of G1 hybrids included in TRS. Adding 354 G1 hybrids to G0R TRSs, increased predictive abilities on average by 0.13 for DMY, 0.20 for DMC, 0.21 DtSilk, and 0.12 for MFU. For TRSs comprising G0R and G0S hybrids, adding 354 G1 hybrids led to smaller gains (gain not exceeding 0.07 for MFU). The largest increase in predictive abilities when updating the TRS with G1 hybrids was obtained with the smallest initial G0R TRS (G0R_F-4H). It is interesting to note that TRSs composed of G0 and 354 G1 hybrids always outperformed prediction accuracies obtained with 354 G1 (cross-validations within G1), illustrating the benefit of keeping information from the previous generation in the TRS.

From Fig. 6, it is possible to estimate the number of G1 hybrids to add to the initial G0R TRSs to achieve similar predictive abilities to the ones obtained when adding 132 G0S hybrids. For example, for DMY and the G0R_F-4H initial TRS, adding 132 G0S was equivalent to adding around 170 G1 hybrids. For all initial G0R TRSs, the number of G1 hybrids to include to be more efficient than 132 G0S hybrids was higher than 132 for all traits except MFU.

### Scenario 3b-optimization of the composition of the factorial TRS for G1 hybrid predictions

**Fig. 7:**
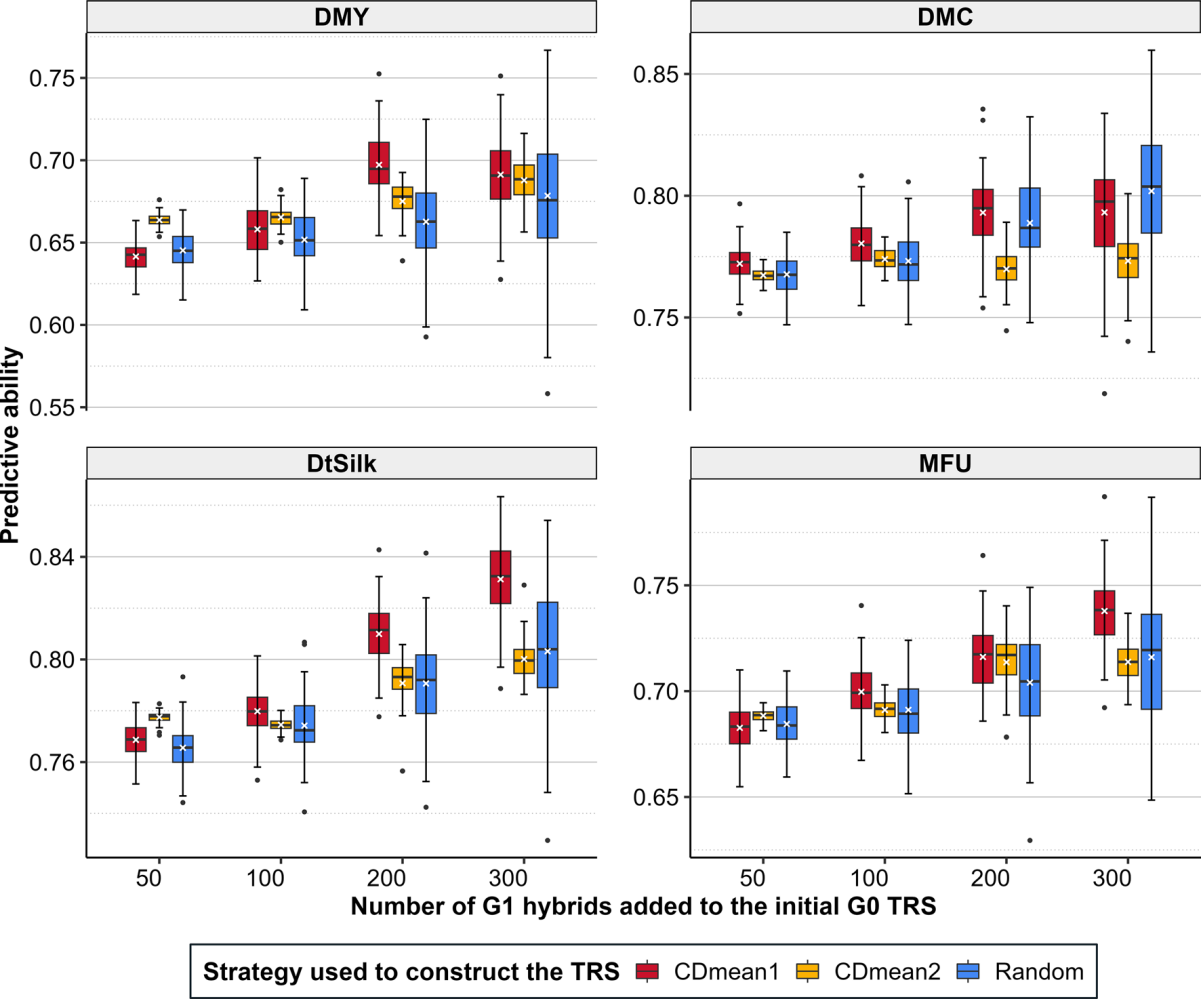
Predictive abilities obtained with TRSs composed of an initial G0 set (1360 hybrids) completed by a CDmean optimized G1 hybrid set of different sizes (50, 100, 200 and 300). The G1 hybrid set is optimized considering only G1 information (CDmean 1) or considering G1 and G0 information (CDmean 2) in the calculation of the CDmean and compared to a randomly sampled TRS (Random). The white cross represents the mean predictive ability over the 100 replicates

The G1 hybrid set to add to the existing G0 TRS (1360 G0 hybrids) was optimized using the CDmean following two strategies, and the results were compared to a TRS obtained from random sampling (Fig. 7). In the first strategy (CDmean1), the G1 hybrid set was optimized without considering the information from the G0 hybrids, whereas in the second strategy (CDmean2), the information from the G0 hybrids was considered. For all traits and all sampling sizes, the best CDmean strategy gave higher or at least equivalent predictive abilities compared to random sampling except for DMC for a sampling size of 300. The maximum gains were 0.03 for DMY, 0.01 for DMC, 0.03 for DtSilk, and 0.02 for MFU, depending on the sampling size. Across the 100 replicates, the variance of the predictive abilities was always lower using the CDmean (1 or 2) than the random sampling. The CDmean1, which does not consider G0 hybrid information to optimize the G1 hybrid set included in the TRS, outperformed the CDmean2 except for small sampling sizes (size 50 and 100 for DMY, size 50 for DMC, DtSilk, and MFU).

## Discussion

### SCA variance and its importance in hybrid breeding

The SCA variance estimated in the G1 generation was small or equal to zero (Tables 3, S2). Small SCA variance was expected in hybrids produced by crossing lines from divergent populations (Reif et al. 2007). The estimated SCA percentage decreased for all traits from G0 to G1 hybrids (Table S3). The precision of SCA variance estimation in our experiment is limited and does not allow us to draw a final conclusion on this evolution. However, one possible explanation for the decrease in SCA variance we observed is that the recurrent reciprocal selection increased the divergence between groups (as also observed by Gerke et al. 2015) and, as a result, decreased the SCA variance in the flint-dent single-cross hybrids (consistent with theoretical expectations from Reif et al. 2007 and Legarra et al. 2023 and simulations from Melchinger and Frisch 2023).

### Genetic gain after selection based on genomic predictions trained on a sparse factorial design

The population was selected for an index combining yield performance (DMY), dry matter content (DMC), and digestibility (MFU) based on genomic predictions. We successfully improved the mean performance of the new generation for DMY, but there was a decrease for MFU and DMC (Table 4), which was higher than expected. The negative correlation (-0.53) between DMY and MFU that was observed based on phenotypic data in the G0 generation (G0_F-1H) (Fig. S4) certainly explains the difficulty of improving both traits simultaneously. This was consistent with results found by Barrière and Emile (2000) and Surault et al. (2005), who also reported a negative correlation of -0.5 between these traits for maize silage. To maintain a stable level of DMC and improve MFU in the new generation, higher weights relative to DMY should have been put on these traits in the index calculation.

The genetic gain predicted by the GBLUP model trained on the G0_F-1H design was similar to the observed genetic gain for DMY. This illustrates the efficiency of GS models in predicting GCA values based on a sparse factorial TRS and confirms the results found by Seye et al. (2020) using simulations and Lorenzi et al. (2022) on the G0 generation.

### Predictive ability in the new generation and comparison of different GS models

Three main prediction scenarios were considered, each corresponding to a realistic breeding program application. As mentioned in the introduction, one main objective for a breeder is to identify the most promising hybrids for varieties creation. Due to limited phenotyping resources, only some of the candidate hybrids are phenotyped, making the genomic prediction of untested hybrids critical for hybrid selection. Scenario 1 falls within this framework. It involves using hybrids between candidate lines from a given generation as TRS of a GS model to predict all remaining hybrid combinations. In Scenario 1, we evaluated the predictive ability within G1 hybrids and compared different prediction models. All models gave high predictive abilities, with the lowest reaching 0.66 (for DtSilk with the PBLUP model). The high predictive ability of the PBLUP model indicates that family structure alone could predict part of hybrid performances. However, GBLUP models always outperformed the PBLUP, confirming the efficiency of GBLUP to predict the mendelian sampling within a family, which is of main interest for breeding. Different GBLUP models were tested. Differences were sometimes significant but always small (< 0.01). Including non-additive genetic effects had little or no effect on predictive abilities, which was also reported in studies using data from inter-heterotic group hybrids (Bernardo 1994; Schrag et al. 2006, 2018; Maenhout et al. 2010; Vitezica et al. 2017; González-Diéguez et al. 2021; Lorenzi et al. 2022). Note that the new SCA kinship formula proposed by González-Diéguez et al. (2021) used in model GCA.2 did not improve predictive abilities compared to the one used in the GCA.1 model. This was also observed by Lorenzi et al. (2022) for genomic predictions within the G0 generation. In their simulations, Seye et al. (2020) found an advantage of including SCA in prediction models when SCA explains about 23% of the genetic variance. The small SCA variances estimated in our experimental design are consistent with the fact that including non-additive effects did not improve prediction accuracies.

Assuming a single additive hybrid genetic effect (G models) or additive genetic effects defined according to the allele origin (GCA models) was equivalent in terms of quality of prediction for hybrid performance. This was surprising considering the large differences in GCA variances observed between the two groups and the detection of group-specific QTLs in the G0_F-1H design (Giraud et al. 2017b). The equivalence in terms of prediction accuracy between the G and GCA models was also shown in hybrid populations by Technow et al. (2014), González-Diéguez et al. (2021), and Alves et al. (2019). Even if the GCA model did not outperform the G model, it makes it possible to estimate parental line values and thus select the parental lines of the next cycle, which is less straightforward with a G model. We kept the GCA.1 model for the following genomic prediction scenarios for these reasons.

### Portability of genomic predictions trained on a sparse factorial across breeding cycles

In hybrid breeding schemes, phenotypic evaluation is based on hybrid progeny testing, which is expensive and requires generations of crossing. GS can accelerate the breeding process by identifying the best lines and single-cross hybrids between them based on the new inbred line marker genotypes. Scenario 2a addressed this objective by assessing the predictive ability of the hybrids in the next generation using TRSs composed of hybrids from the previous generation. We trained the GS model on the G0_F-1H to predict G0S and G1 hybrids, allowing us to evaluate the predictive ability across cycles and environments. We obtained high predictive abilities for G0S hybrids, which illustrates the ability of the GS model trained on the G0_F-1H design to predict the performances of a new set of hybrids between selected lines in new environments. This confirms previous results (Lorenzi et al. 2022), which considered another set of G0S hybrids evaluated in the 2016–2017 G0_F-4H trials. We observed lower predictive abilities for G1 compared to G0S hybrids. Note that G0S and G1 hybrids were evaluated in the same environments, therefore, the decrease in predictive ability is not attributable to an environmental effect. A decrease in prediction accuracy when generations differ between the TRS and PS was reported in simulations (Pszczola and Calus 2016; Seye et al. 2020) and experimental studies on hybrids (on sugar beet by Hofheinz et al. 2012; on barley Sallam et al. 2015 and Michel et al. 2016 and on maize by Wang et al. 2020). This decrease is expected as selection modifies allele frequencies along generations, and recombination events modify marker-QTL linkage disequilibrium. Allelic frequencies are identical in G0S and G1 hybrids since G1 lines are the unselected progeny of G0S lines. Thus, the lower predictive ability observed in the G1 compared to the G0S hybrids is due to the recombination events. Still, predictive abilities remained high, highlighting the efficiency of the GS model trained on the G0_F-1H design in decorrelating the contributions from each parental line to predict their GCAs, the GCAs of their G1 progeny, and therefore the hybrid values across breeding cycles.

### Efficiency of factorial compared to tester TRSs for predictions across the breeding cycle

Compared to tester designs, factorials allow the estimation of GCA and SCA components early in the selection process and could prevent the bias due to the use of a small number of testers. It is therefore expected to be more efficient than a tester-based evaluation at a fixed number of hybrids when SCA variance is large (Seye et al. 2020). A previous study using the same TRSs as in the present study to predict the G0 generation showed slightly higher predictive abilities using the factorial compared to the tester TRSs (Lorenzi et al. 2022). This small advantage was likely due to (i) the small SCA variance observed in the G0 generation, (ii) the use of founder lines as testers, reducing the expected differences between tester and factorial designs (as shown by simulation by Seye et al. 2020), and (iii) suboptimal factorial design composition in terms of the number of hybrids per line (Technow et al. 2014 and Lorenzi et al. 2022).

In the present study, we investigated which TRS between factorial and tester was more efficient to predict the next generation (G1) at the same number of hybrids and lines (Scenario 2b). The advantage of the factorial TRS decreased when predicting the new generation (G1) compared to what was found in the G0 generation (Lorenzi et al. 2022). This is in accordance with results from simulations based on a similar design (Seye et al. 2020), which showed that the advantage of the factorial over the tester TRSs decreases across breeding cycles if the TRS is not updated. In Scenario 2b’, we investigated several tester design compositions with different number of lines but the same number of hybrids. As already found in the G0 generation by Lorenzi et al. (2022), we showed that the best strategy was always to use more testers while maximizing the number of candidate lines, a strategy comparable to using a sparse factorial design with a small number of hybrids per line.

### Benefit of updating the factorial TRS along breeding cycles

Once inbred lines from a new generation (G1) are available and can be genotyped, a key issue is to predict the best new hybrid combinations between them to prioritize hybrid production and evaluation. There are two possible situations, depending on the availability of phenotypes of a subset of hybrids from the new breeding cycle (G1 hybrids). When G1 phenotypes are available, they can be used to calibrate prediction equations. We showed the benefit of combining this information with historical data from G0 hybrids compared to using G1 phenotypes alone (Fig. 6). Several studies also reported similar results (Jannink 2010; Denis and Bouvet 2013; Neyhart et al. 2017). Among the historical data, hybrids between the lines selected to generate the new generation (G0S) are the most related to the G1 generation. We showed that even when G0S and G1 hybrids were already in the TRS, there was still a benefit of including hybrids between unselected lines from previous generations (G0R hybrids). This last result aligns with results found by Neyhart et al. (2017) and Brandariz and Bernardo (2018), showing that when constructing a TRS, one must consider keeping hybrids produced between unselected lines to maintain high prediction accuracy. Additionally, when including data from the two generations (G0 and G1) in the TRS, we also included TRS hybrids evaluated in different years and environments. This reduced the impact of genotype-by-environment interactions and, as a result, increased prediction accuracy. Similar results have been obtained by Auinger et al. (2016).

In the second situation, where G1 hybrids phenotypes are not yet available, we showed that using only historical data in the TRS can provide good prediction accuracies (Fig. 6). We evaluated the benefit of producing and phenotyping additional data to update the historical (G0) TRS, particularly the benefit of adding G0S hybrids. G0S are single-crosses between the G0 lines selected to be the parents of the G1 generation, so including these hybrids increases the relationship between the TRS and the G1 PS. We compared G0S and G1 hybrids for their efficiency to update the TRS. Predictive abilities obtained with the 132 G0S hybrids were reached when adding a similar number or more G1 hybrids (Fig. 6). This indicates that for a fixed number of hybrids, using G0S hybrids was equivalent to or slightly better than using G1 hybrids for updating the TRS. Once the best candidate lines are selected to become the parental lines (corresponding to G0S lines) for the subsequent breeding cycle, but hybrids from the new cycle (G1 hybrids) are not yet available, it is beneficial to phenotype new hybrid combinations between the selected lines to update the TRS. Several entangled factors can explain the result: (i) the increased TRS size, (ii) the increased relationship between the TRS and PS, and (iii) the increased number of years and environments in the data used as TRS (see reviews by Isidro y Sánchez and Akdemir 2021 and Rio et al. 2022a, b). Adding G0S hybrids is a way to accumulate information on the hybrid values (GCAs) of the selected lines in different environments, which is helpful to predict the hybrid values of their progeny. Our results also show that even if G0S hybrids are added to the TRS, it is still interesting to add performances of G1 hybrids to the TRS when these become available, as it increases the genotypic relatedness between the TRS and the PS (Fig. 6).

### Optimization of the G1 hybrid set to phenotype to update the TRS

As phenotyping capacities are constraint, it is important to optimize the set of hybrids from the new generation to be added to the TRS. This was done in Scenario 3b, where we optimized the G1 hybrid set used to update the initial G0 TRS. The G1 hybrid set was optimized based on the CDmean computed considering (CDmean 2) or not (CDmean 1) the information from the initial G0 TRS (G0R + G0S hybrids) (Fig. 7). As expected, optimizing the TRS using the CDmean (CDmean 1 or CDmean 2) instead of random sampling increased our predictive abilities in most of the cases. This was also reported in numerous other studies (Rincent et al. 2012, 2017; Isidro et al. 2015; Akdemir et al. 2015; Mangin et al. 2019; Isidro y Sánchez and Akdemir 2021; Kadam et al. 2021). Interestingly, we observed more stable predictions abilities across replicates using the CDmean, than with random sampling. It has to be noted that in this optimization process, we used CDs computed assuming a single additive genetic effect despite using a GCA/SCA prediction model for our predictions. We could have included non-additive effects in the computation of the CDmean, as done by Momen and Morota (2018) and Fristche-Neto et al. (2018). However, these authors did not find a clear benefit of accounting for dominance in the CDmean computation, and we did not see any advantage of including the dominance effect in our prediction models. For these reasons, we do not expect that adding dominance in the CDmean computation would have had a positive impact.

It was surprising to us that the CDmean 1 (which does not consider information from the G0 TRS hybrids) outperformed the CDmean 2 (which considers the G0 information). The CDmean 1 likely selected G1 hybrids that were representative of the whole range of G1 hybrids. In contrast, since the hybrids between the G0S parental lines of the G1 were already in the TRS, the CDmean 2 likely maximized the diversity of the TRS by favoring G1 hybrids genetically distant from the G0 hybrids. The CDmean 2 assumed that G0 and G1 hybrids were evaluated in the same environments, which was not true. As a consequence, some of the G0 hybrids may not have been as informative to predict the G1 hybrids as they seemed, based on the genomic relationship matrix. This may explain why CDmean 2 did not outperform CDmean 1. To compute the CDmean, we could have considered each environment as a different trait and used the correlation value between the two environments, as suggested by Ben-Sadoun et al. (2020). Rio et al. (2022a) showed the benefit of using such multi-environmental CDs to optimize the allocation of individuals in trial networks, and this could have been extended to multigeneration TRS optimization. In practice, one cannot know in advance the correlation between the environments where the previous generation was evaluated and those where the new generation will be evaluated. One solution might be to use historical data to estimate the magnitude of correlations that can be expected between years and use this value when computing the expected multi-environment CD.

## Conclusions

Our study confirms the efficiency of combining genomic predictions and sparse factorial TRS to predict candidate lines GCAs and hybrid values across breeding cycles. Genomic prediction accuracy was high and increased when updating the TRS by incorporating performances of hybrids between selected lines from the previous generation and potential hybrids from the new generation. When incorporating hybrids from the new generation, choosing them based on a criterion such as the CDmean was beneficial.

### Supplementary Information

Below is the link to the electronic supplementary material.

Supplementary file1 (PDF 546 KB)

## Data Availability

Data from the first generation were already published as supplementary materials of a previous paper (Giraud et al. 2017b). The datasets generated during and/or analyzed during the current study and the description of the data are available in the INRAE Dataverse repository, https://doi.org/10.57745/VISRTZ.
