# Supplementary material for: Portability of genomic predictions trained on sparse factorial designs across two maize silage breeding cycles
Source: Theor Appl Genet. 2024 Mar 7;137(3):75. doi: 10.1007/s00122-024-04566-4 (PMC11341662; doi:10.1007/s00122-024-04566-4)
Supplement: Supplementary file 1 — Supplementary file1 (PDF 546 KB) [file 122_2024_4566_MOESM1_ESM.pdf]

Portability of genomic predictions trained on sparse factorial designs across two maize silage breeding cycles

Alizarine Lorenzi[1*], Cyril Bauland[1], Sophie Pin[1], Delphine Madur[1], Valérie Combes[1], Carine Palaffre[2], Colin Guillaume[3], Gaëtan Touzy[4], Tristan Mary-Huard[1,5], Alain Charcosset[1], Laurence Moreau[1]

[1] Université Paris-Saclay, INRAE, CNRS, AgroParisTech, Génétique Quantitative et Evolution - Le Moulon, 91190 Gif-sur-Yvette, France
[2] UE 0394 SMH, INRAE, 2297 Route de l'INRA, 40390, Saint-Martin-de-Hinx, France
[3] Maïsadour Semences SA, F-40001 Mont-de-Marsan Cedex, France
[4] RAGT2n, Genetics and Analytics Unit, 12510 Druelle, France
[5] MIA, INRAE, AgroParisTech, Université Paris-Saclay, 75005, Paris, France

Corresponding author: Laurence Moreau laurence.moreau@inrae.fr. *ORCID:* 0000-0002-7195-1327

Supplementary materials

File S1-Spatial corrections

The BLUPs of spatial effects were estimated on raw performance using model (S1) on the (G0S+G1)_F-1H design. The model implemented was:

$$Y_{hii'lxyz} = \mu + \lambda_l + \tau_h \times t_h + H_{h(ii')} \times (1 - t_h) + \left(R_{x(l)} + C_{y(l)}\right) \times (1 - d_l) + B_{z(l)} \times d_l + E_{hii'lxyz}, \quad \text{(S1)}$$

where $Y_{hii'lxyz}$ is the phenotypic value of hybrid h produced by crossing the parental lines i and i', evaluated in environment, l located at row x, column y and in block z. μ is the intercept, λ_l is the fixed effect of environment l, t_h distinguishes the type of hybrid, it is set to 0 for the experimental hybrids and set to 1 for the controls, τ_h is the vector of fixed effect of the control with 18 levels (two for the commercial hybrids and 16 for the founder controls). With $H_{h(ii')}$ the random genetic effect of hybrid h produced by crossing the flint line i and the dent line i', defined as follows: $H_{h(ii')} = U_i + U'_{i'} + S_{ii'}$, where U_i (respectively $U'_{i'}$) is the random GCA effect of the flint line i (respectively dent line i'), we assume that U_i ($U'_{i'}$) are independent and identically distributed (iid) and follow a normal distribution: $U_i \sim \mathcal{N}(0, \sigma^2_{GCA_f})$ (and $U'_{i'} \sim \mathcal{N}(0, \sigma^2_{GCA_d})$). $S_{ii'}$ is the random SCA effect of the interaction between the parental lines i and i', with $S_{ii'} \sim \mathcal{N}(0, \sigma^2_{SCA})$ idd. $R_{x(l)}$ is the random effect of row x in environment l, $C_{y(l)}$ is the random effect of column y in environment l and $B_{z(l)}$ is the random effect of block z in environment l, with $R_{x(l)} \sim \mathcal{N}\left(0, \sigma^2_{R_l}\right)$, $C_{y(l)} \sim \mathcal{N}(0, \sigma^2_{C_l})$ and $B_{z(l)} \sim \mathcal{N}(0, \sigma^2_{B_l})$ which are assumed to be independent and identically distributed (iid). d_l indicates the spatial effect to consider in environment l, it is set to 1 if the spatial effect is modeled by a block effect and by 0 if modeled by row and column effects. To construct the vector d, for each trait and each trial, two types of spatial effects were tested the line and column effects or the block effect. The best spatial effect was chosen based on the AIC criterion. $E_{hii'lxyz}$ is the error term of the model, with $E_{hi'lxyz} \sim \mathcal{N}\left(0, \sigma^2_{E_l}\right)$ iid within trial and independent between trials. The different random effects of the model are assumed to be independent.

Table S1 Broad-sense heritability (H²), percentage of genetic variance assigned to SCA variance (%SCA) and variance components estimated on phenotypic data corrected for spatial effects for the (G0S+G1)_F-1H without marker information using (Model 1).

**Trait**	**Hybrid**	$\sigma^2_{GCA_f}$	$\sigma^2_{GCA_d}$	σ^2_{SCA}	$\sigma^2_{GCA_f xE}$	$\sigma^2_{GCA_d xE}$	σ^2_{SCAxE}	σ^2_E **min-max**[a]	**%SCA**[b]	**H² [c]**
**DMY**	**G0S**	0.50(0.24)	0.13(0.18)	0.14(0.18)	0.07(0.08)	0.12(0.09)	0.00	0.31(0.05)-1.40(0.12)	19	0.87
	**G1**	0.31(0.05)	0.25(0.05)	0.00	0.05(0.05)	0.07(0.04)	0.23(0.07)		0	0.80
**DMC**	**G0S**	0.46(0.28)	1.42(0.51)	0.00	0.00	0.39(0.13)	0.00	0.57(0.07)-3.62(0.27)	0	0.90
	**G1**	1.47(0.21)	0.73(0.21)	0.25(0.20)	0.11(0.05)	0.31(0.05)	0.00		10	0.92
**DtSilk**	**G0S**	1.63(0.71)	1.57(0.65)	0.03(0.27)	0.41(0.16)	0.05(0.12)	0.00	0.64(0.09)-2.33(0.20)	1	0.93
	**G1**	2.06(0.29)	1.41(0.27)	0.05(0.22)	0.17(0.07)	0.09(0.06)	0.24(0.11)		1	0.94
**PH**	**G0S**	13.30(25.69)	32.70(29.46)	45.20(33.71)	10.16(9.38)	15.22(10.15)	0.00	31.30(4.11)-108.33(9.08)	50	0.88
	**G1**	32.11(5.80)	51.45(7.13)	0.00	3.38(3.24)	0.00	15.81(4.78)		0	0.88
**DINAG**	**G0S**	0.71(0.37)	0.00	0.08(0.29)	0.32(0.45)	0.00	0.78(0.54)	1.47(0.20)-5.37(0.44)	10	0.56
	**G1**	0.48(0.12)	0.78(0.14)	0.00	0.16(0.15)	0.17(0.15)	0.55(0.26)		0	0.68
**DINAGZ**	**G0S**	0.51(0.31)	0.00	0.00	0.74(0.45)	0.00	0.42(0.43)	1.08(0.14)-3.88(0.35)	0	0.43
	**G1**	0.47(0.17)	0.65(0.16)	0.06(0.19)	0.14(0.15)	0.21(0.15)	0.44(0.25)		5	0.66
**MFU(x10²)**	**G0S**	0.44(0.24)	0.00	0.19(0.13)	0.27(0.17)	0.06(0.12)	0.00	0.12(0.02)-7.97(0.55)	30	0.56
	**G1**	0.49(0.09)	0.36(0.09)	0.00(0.09)	0.20(0.07)	0.13(0.07)	0.11(0.09)		0	0.62
**NDF**	**G0S**	0.46(0.22)	0.00	0.16(0.13)	0.01(0.13)	0.12(0.13)	0.00	0.11(0.00)-5.49(0.38)	25	0.62
	**G1**	0.52(0.09)	0.33(0.09)	0.06(0.09)	0.09(0.06)	0.05(0.06)	0.10(0.08)		6	0.70
**LIGN(x10²)**	**G0S**	0.33(0.67)	1.42(0.82)	0.00	2.79(1.31)	1.09(1.16)	0.21(0.78)	1.02(0.16)-23.93(1.84)	0	0.47
	**G1**	1.16(0.31)	0.77(0.27)	0.00	2.45(0.47)	1.75(0.46)	0.66(0.59)		0	0.48
**CELL(x10²)**	**G0S**	3.81(3.08)	5.12(2.86)	0.00	0.00(2.00)	0.00	0.00	2.73(0.38)-505.18(32.48)	0	0.30
	**G1**	1.63(0.99)	3.61(0.99)	1.28(1.10)	1.23(0.69)	0.00	0.00		20	0.24
**HCELL(x10²)**	**G0S**	3.26(3.16)	6.65(3.62)	0.12(1.81)	1.32(2.78)	0.00	0.00	2.60(0.39)-418.84(27.37)	1	0.30
	**G1**	6.18(1.61)	3.66(1.60)	1.25(1.61)	3.58(1.42)	1.28(1.22)	0.00		11	0.32

[a] Minimum and maximum residual variance across all environments

[b] Percentage of SCA variance computed as $\frac{\sigma^2_{SCA}}{\sigma^2_{GCA_d}+\sigma^2_{GCA_f}+\sigma^2_{SCA}} \times 100$

[c] Broad-sense heritability

[d] Standard error in bracket

Table S2 Performances (ls-means) of commercial, founder and experimental hybrids (G0S and G1 hybrids) and genetic gain of the experimental hybrids compared to the founder hybrids corresponding to the (G0S+G1)_F-1H design.

Trait	Ls-means												Genetic Gain[b]	
	Commercial[a]			Founder[a]			G0S[a]			G1[a]			G0S[a]	G1[a]
	Mean	Min-Max	Sd[c]	Mean	Min-Max	Sd	Mean	Min-Max	Sd	Mean	Min-Max	Sd		
**DMY**	**17.96**	17.18-18.74	1.10	**15.80**	14.27-17.47	0.84	**17.35**	14.82-18.89	0.92	**17.33**	14.33-19.72	0.85	1.55	1.52
**DMC**	**34.69**	34.37-35.00	0.45	**34.10**	30.53-37.48	1.94	**33.33**	30.54-37.13	1.40	**33.43**	29.39-39.18	1.63	-0.77	-0.67
**DtSilk**	**201.89**	200.27-203.50	2.28	**203.14**	199.80-206.16	1.50	**204.97**	201.80-208.79	1.82	**205.04**	197.98-211.40	1.97	1.83	1.90
**PH**	**246.31**	239.86-252.76	9.12	**245.11**	222.10-279.19	13.44	**259.52**	235.54-283.59	10.40	**257.50**	227.27-286.97	10.15	14.41	12.40
**DINAG**	**51.14**	50.71-51.57	0.61	**51.03**	47.48-54.84	2.09	**49.73**	47.54-52.63	1.35	**49.61**	45.08-53.35	1.37	-1.30	-1.42
**DINAGZ**	**43.13**	42.56-43.70	0.81	**42.98**	39.75-46.35	1.91	**42.22**	39.81-45.17	1.29	**42.03**	38.12-45.80	1.36	-0.75	-0.94
**MFU**	**95.54**	95.34-95.74	0.28	**95.28**	91.13-98.14	2.05	**93.17**	89.04-97.06	1.56	**93.13**	87.59-101.36	1.71	-2.11	-2.15
**NDF**	**41.10**	40.73-41.48	0.53	**41.34**	39.88-42.16	0.63	**42.07**	39.19-43.94	0.95	**42.11**	38.27-46.39	1.12	0.73	0.77
**LIGN**	**5.41**	5.37-5.46	0.07	**5.73**	5.27-6.41	0.32	**5.86**	5.20-6.26	0.23	**5.89**	5.28-7.00	0.23	0.13	0.17
**CELL**	**51.76**	51.51-52.01	0.35	**51.64**	50.85-52.40	0.50	**52.07**	50.92-53.34	0.48	**52.10**	50.55-53.91	0.46	0.44	0.46
**HCELL**	**42.90**	42.50-43.30	0.57	**42.70**	41.50-43.56	0.72	**42.04**	40.69-43.56	0.61	**42.01**	39.64-43.97	0.63	-0.66	-0.69

[a] Hybrid type in the experimental design comprise hybrids used as controls (commercial or founder hybrids) and experimental hybrids from the first generation (G0S) or the new generation (G1)
[b] Genetic gain computed as the difference between the mean performance of the experimental hybrids and the mean performance of the founder hybrids
[c] Standard deviation

Table S3 Broad-sense heritability (H^2), percentage of genetic variance assigned to SCA variance (%SCA) and variance components estimated on phenotypic data corrected for spatial effects for the (G0S+G1)_F-1H without marker information (Model 1).

**Experimental design**	**Component**	**DMY**	**DMC**	**DtSilk**	**PH**	**DINAG**	**DINAGZ**	**MFU**	**NDF**	**LIGN**	**CELL**	**HCELL**
**G0_F-1H**	H^2	0.84	0.91	0.91	0.90	0.85	0.79	0.79	0.72	0.89	0.78	0.84
	%SCA	20	15	17	15	0	0	10	13	0	3	0
**G0_F-4H**	H^2	0.89	0.93	0.93	0.92	0.87	0.78	0.86	0.80	0.86	0.57	0.73
	%SCA	9	6	6	3	2	4	3	6	0	3	3
**G0_T-D**	H^2	0.86	0.86	0.84	0.90	0.87	0.79	0.81	0.68	0.82	0.55	0.72
	%SCA	20	14	11	5	5	3	11	25	8	15	12
**G0_T-F**	H^2	0.85	0.93	0.87	0.87	0.77	0.67	0.75	0.76	0.69	0.36	0.44
	%SCA	21	13	17	31	14	19	14	18	0	13	11
**(G0S+G1)_F-1H**	H^2	0.81	0.92	0.94	0.88	0.68	0.65	0.62	0.70	0.48	0.24	0.32
	%SCA	0	7	1	0	0	1	7	9	0	17	9
**G1_F-1H**	H^2	0.80	0.92	0.94	0.88	0.69	0.66	0.62	0.70	0.48	0.23	0.32
	%SCA	0	10	1	0	0	4	0	6	0	19	11

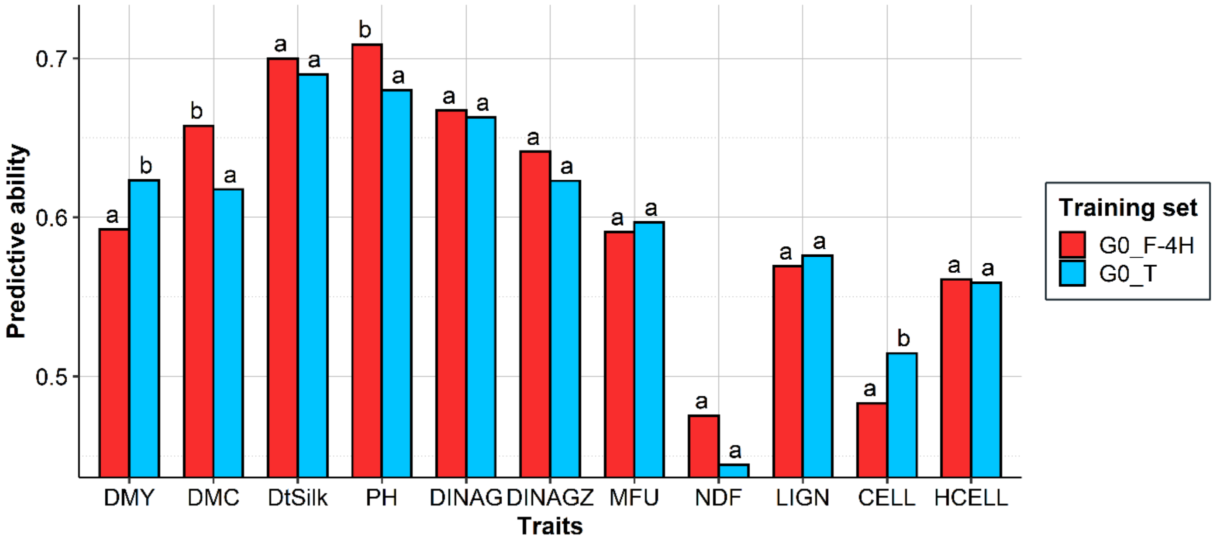

Fig.S1 Predictive abilities obtained for the G1 hybrids (442) by training the GS model on the G0_F-4H (363) or the G0_T (360) TRSs in Scenario 2b. Williams tests were performed (α=0.05) and significant differences were indicated with letters: two different letters indicate a significant difference and at least one common letters indicate no significant difference.

Table S4 Correlation between thepredicted G1 GCA line values or G1 hybrid values obtained using the factorial and the tester designs as TRS.

Traits	Correlation between tester- and factorial-based GCA BLUPs		Correlation between tester- and factorial-based predicted hybrid values
	Dent	Flint	
**DMY**	0.90	0.84	0.87
**DMC**	0.85	0.92	0.87
**DtSilk**	0.86	0.94	0.91
**PH**	0.90	0.77	0.85
**DINAG**	0.94	0.83	0.91
**DINAGZ**	0.93	0.79	0.90
**MFU**	0.94	0.85	0.89
**NDF**	0.86	0.78	0.81
**LIGN**	0.89	0.83	0.87
**CELL**	0.88	0.80	0.85
**HCELL**	0.91	0.81	0.88

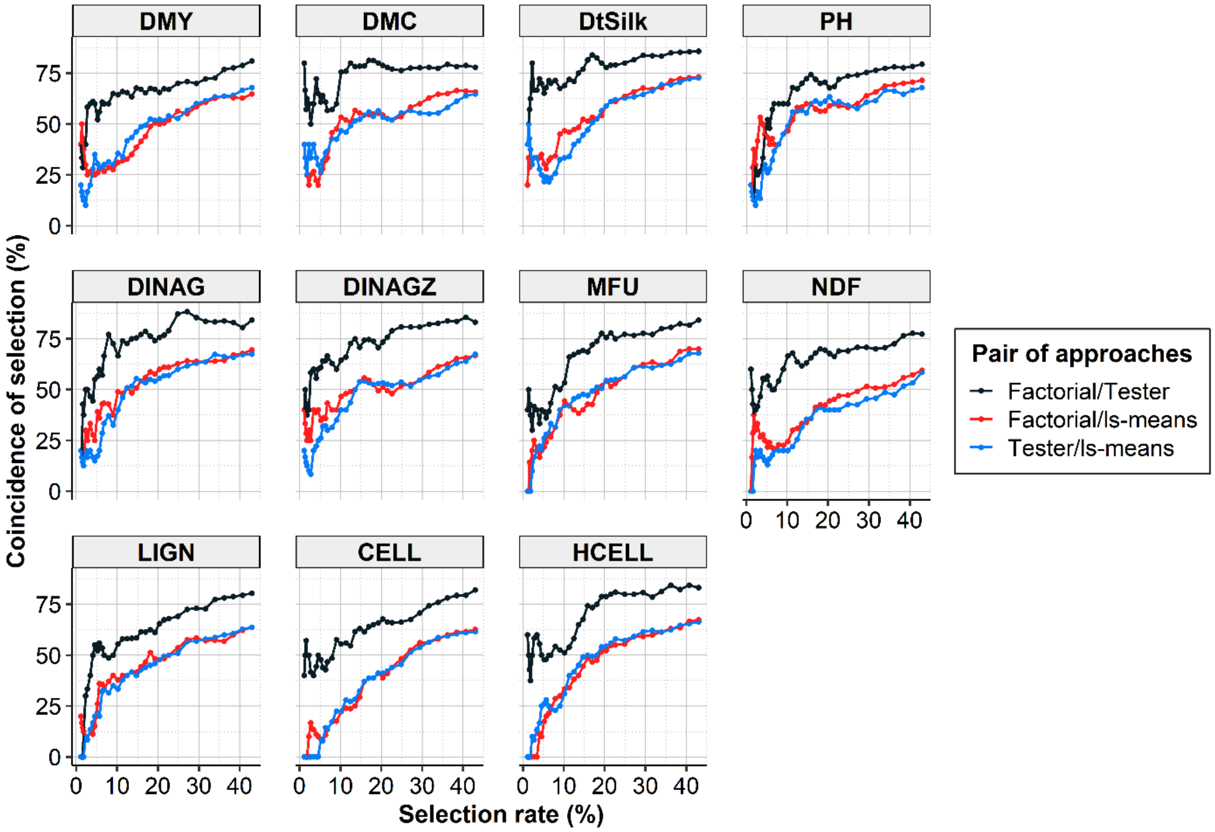

Fig.S2 Coincidence of selection of the G1 single-cross hybrids (442) computed for different selection rates (%). The coincidence of selection was computed in black between the factorial and the tester approaches (GBLUP), in red between the factorial approach (GBLUP) and the phenotypic values (hybrid ls-means) and in blue between the tester approach (GBLUP) and the phenotypic values (hybrid ls-means). The BLUPs estimated in Scenario 2a (Figure S1) were used to compute the coincidence of selection.

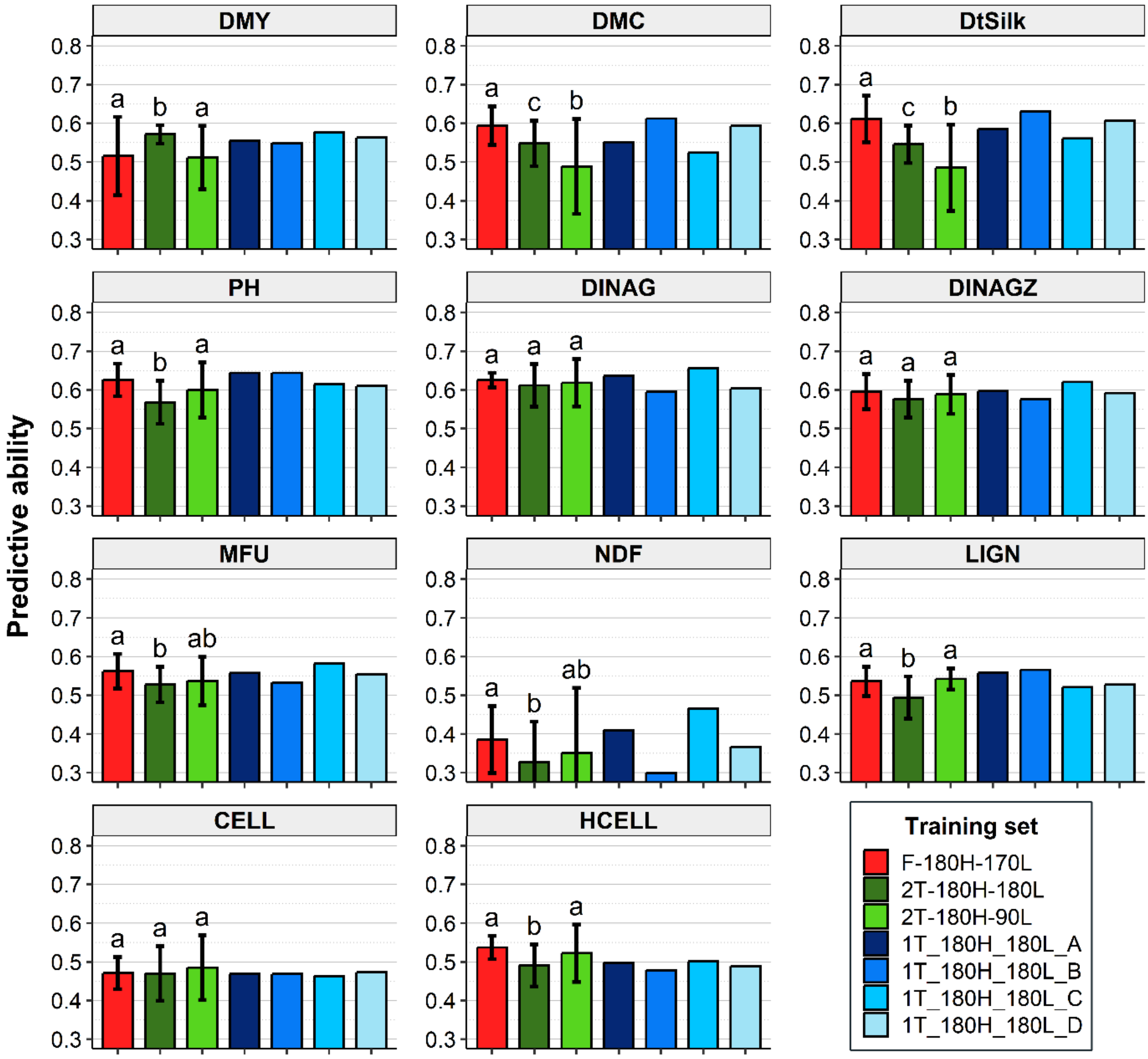

Fig.S3 Predictive abilities obtained in Scenario 2b' by training the GS model on 180 hybrids issued from tester-based or factorial TRSs to predict the G1 hybrids (442). The different tester-based TRSs correspond to: 90 lines crossed to one tester (1T-180H-180L-A, 1T-180H-180L-B, 1T-180H-180L-C, 1T-180H-180L-D), 90 lines crossed to two testers (2T-180H-180L), 45 lines crossed to two testers (2T-180H-90L). The factorial design (F-180H-152L) corresponds to the crosses of 76 flint lines with 76 dent lines. The sampling was repeated 10 times and t-tests (α=0.05) were performed for the F-180H-170L, 2T-180H-180L and 2T-180H-90L. Significant differences were indicated with letters: two different letters indicate a significant difference and at least one common letters indicate no significant difference.

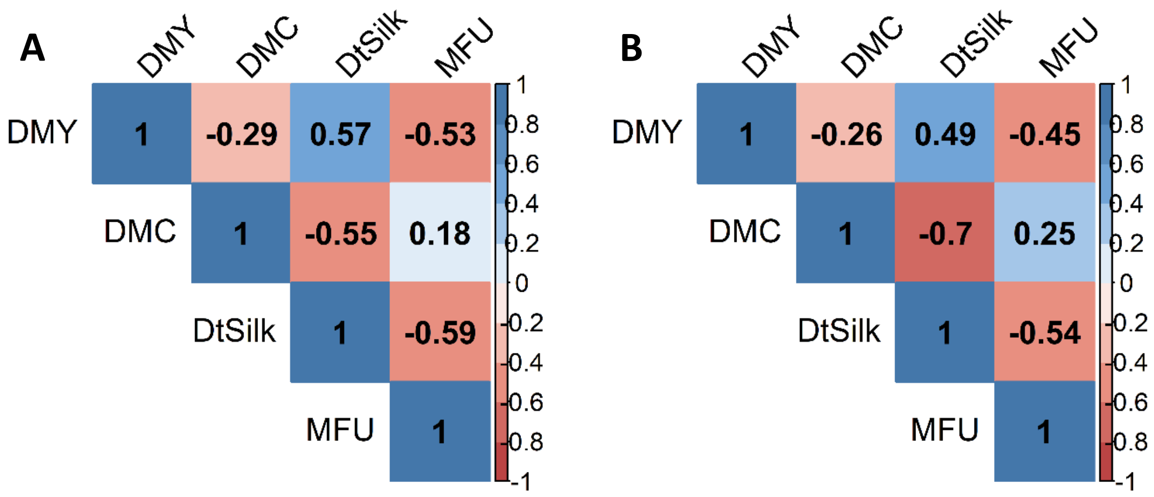

Fig.S4 Phenotypic correlation between traits in the G0_F-1H design (A) or in the G1 hybrids (B).
